# Isolation and transformation of perennial ryegrass (*Lolium perenne* L.) protoplasts for the *in vivo* assessment of guide RNAs editing efficiency

**DOI:** 10.3389/fpls.2025.1744085

**Published:** 2026-01-16

**Authors:** Ferenz Sustek-Sánchez, Erki Eelmets, Lenne Nigul, Kairi Kärblane, Martin Laasmaa, Madara Balode-Sausina, Sanda Astra Berzina, Davis Ducis, Elza Kaktina, Kristina Jaškūne, Odd Arne Rognli, Nils Rostoks, Cecilia Sarmiento

**Affiliations:** 1 Department of Chemistry and Biotechnology, Tallinn University of Technology, Tallinn, Estonia; 2 Department of Cybernetics, Tallinn University of Technology, Tallinn, Estonia; 3 Department of Molecular Life Sciences, Microbiology and Biotechnology, Faculty of Medicine and Life Sciences, University of Latvia, Riga, Latvia; 4 Laboratory of Genetics and Physiology, Lithuanian Research Centre for Agriculture and Forestry, Akademija, Lithuania; 5 Department of Plant Sciences, Faculty of Biosciences, Norwegian University of Life Sciences (NMBU), Ås, Norway

**Keywords:** CRISPR-Cas, gene editing, guide RNA, *Lolium perenne* L., PEG, perennial ryegrass, polyethylene glycol, protoplasts

## Abstract

Protoplasts are broadly used to perform different cellular and genetic assays. Transformation of protoplasts requires isolation methods that generate a large number of intact cells suitable for downstream applications. *Lolium perenne* L. is an important forage grass species for which gene editing techniques are in their early stages. Using protoplasts has previously been reported as a suitable approach to test the genome editing efficiency of guide RNAs in important grass species like wheat and rice. This approach can speed up and increase the chances of generating edited plants, especially when working with species for which stable transformation methods have not been established yet. Testing two different approaches regarding the processing of *L. perenne* L. tillers showed that using a blender for disintegrating the tissue was easier and faster than cutting the tillers with a razor blade. Conversely, the more classical strategy (cutting with a razor) provided a higher number of viable protoplasts. The use of an enzyme solution containing 2% cellulase during 8 h was shown to be the best experimental condition for protoplast isolation. The addition of a sucrose cushion improved the purification of alive cells, which were then positively transformed with guide RNA encoding vectors using polyethylene glycol. The presence of indels induced by these vectors was then confirmed through decomposition-based analysis of their sequenced genomic DNA. These results demonstrated the suitability of using protoplasts for the *in vivo* assessment of guide RNAs editing efficiency.

## 1 Introduction

The rapidly growing population demands safe food and feed and thus the need for the development of high-yielding crops adaptable to future climate conditions. In temperate regions, climate change may provide the opportunity to expand the list of cultivated forage crops for livestock farming, but on the other hand it may compromise already cultivated species (McEvoy et al., 2011; Grinberg et al., 2016; Kemešytė et al., 2017, Kemešytė et al., 2023). Perennial ryegrass (*Lolium perenne* L.) is the predominant forage grass species in livestock farming (Humphreys et al., 2010), due to its quick establishment, robust regrowth, and high nutritional value for ruminants (Wilkins, 1991). However, perennial ryegrass performs poorly under adverse environmental conditions compared to other cool‐season forage grass species, compromising its cultivation of it in northern-eastern regions of Europe (Kemešytė et al., 2017; Cyriac et al., 2018). Thus, it is of vital importance to develop crops with high, stable, and good quality yields. The development of new cultivars of outcrossing species using traditional breeding techniques is a long and time-consuming process (Pfender, 2009; Sampoux et al., 2011; Sustek-Sánchez et al., 2023).

Moreover, this reproductive system makes *L. perenne* a highly heterogeneous and heterozygous species, which creates an additional challenge when performing genetic studies. Forward-genetics approaches used in crops to identify e.g., abiotic stress resistance genes cannot be easily applied in perennial ryegrass. Genome editing using CRISPR-Cas systems offers an alternative solution for identifying mechanisms underlying stress tolerance and achieving increased frost and drought tolerance of *L. perenne* (Sustek-Sánchez et al., 2023). Nevertheless, when generating edited plants, there is usually a need for the induction, propagation, transformation, and regeneration of calli, making the process time-consuming and complex. Furthermore, in some important species like perennial ryegrass, the generation, transformation, and regeneration of calli has either not been achieved or is in the early stages of methods development with each of the above steps being highly genotype dependent. To date, few publications describe induction of CRISPR-Cas9-targeted mutations in *L. perenne*, all of which used embryogenic calli for either biolistic (Zhang et al., 2020) or *Agrobacterium*-mediated transformation (Grogg et al., 2022; Kumar et al., 2022). The scarcity of available data thoroughly evaluating the impact of each protocol step slows down the development of robust, efficient, and genotype-independent methods for the transformation of *L. perenne*. Screening plasmids containing the Cas9 gene and gRNA arrays for gene editing efficiency prior to their use in calli transformation, makes the process of obtaining edited perennial ryegrass genotypes more time and resource efficient.

The design of guide RNAs (gRNAs) relies generally on bioinformatic tools that predict how efficient and specific a guide is expected to be. However, the efficiency of a gRNA predicted using in silico tools often does not agree with experimental data (Bennett et al., 2020; Konstantakos et al., 2022). This showcases the need for empirically testing the gRNAs *in vivo*, to evaluate their ability to generate indels.

The transformation of protoplasts is an efficient method to perform diverse cellular, molecular, and genetic studies (Eeckhaut et al., 2013; Wu and Hanzawa, 2018). They have also been proved useful in evaluating the efficiency of gRNAs and editing reagents to generate genome-edited plants (Shan et al., 2013; Nadakuduti et al., 2019; Brandt et al., 2020; Yue et al., 2021; Hu and Huang, 2022). Protoplasts can be isolated by millions, which provides an excellent tool for testing genome editing applications. Genomic DNA from transformed protoplasts can be analyzed by sequencing PCR-amplified regions of interest. The application of decomposition-based tools, such as TIDE, allows the identification of edited cells and thus the evaluation of genome editing efficiency (Brinkman et al., 2014).

In this study, we aimed at establishing a reproducible method that would allow *in vivo* evaluation of the editing efficiency of specific gRNAs and transformation vectors using protoplasts. While similar approaches have been used in other grasses, reproducible protocols are lacking for *Lolium perenne*. For this, two different protoplasts isolation methods were studied and compared to determine the best performing technique, yielding a high number of protoplasts whose quality would make them suitable for downstream applications such as polyethylene glycol (PEG)-mediated transformation. Two different genes were used as targets for the evaluation of editing efficiency of binary vectors. *CBP20* is a gene related to cuticular wax accumulation, which has been used to generate drought tolerant barley plants by knocking it out (Daszkowska-Golec et al., 2017, 2020). *CRPK1* is a known negative regulator of proteins resulting from the activation of the ICE-CBF-COR pathway by ABA-dependent responses (Liu et al., 2017; Pashapu et al., 2024).

The DNA of transformed cells can be sequenced and analyzed using decomposition methods to identify and evaluate the presence of indels. This can be used to assess the editing efficiency of reagents such as binary vectors coding for specific gRNAs. In organisms recalcitrant to common transformation methods, such as perennial ryegrass via Agrobacterium-mediated transformation, the described *in vivo* screening can accelerate the generation of edited plants.

## 2 Material and methods

### 2.1 Plant material

The plant material was obtained from germinated seeds of the *Lolium perenne* cv. Veja. and from *in vitro* propagated tillers of a freezing sensitive perennial ryegrass genotype (previously characterized in Pashapu et al., 2024). Plant material from cv. Veja was used to test the different parameters of the protoplasts isolation procedure and to perform the transformations aimed at editing the perennial ryegrass *CBP20* gene (*LpCBP20*). Plant material from a freezing sensitive genotype was used for the transformation experiments to edit the *CRPK1* gene of *L. perenne* (*LpCRPK1*).

The seeds were surface sterilized by washing them with 70% ethanol (3 min), commercial bleach (5% sodium hypochlorite, 90 min), followed by autoclaved distilled water (3 to 5 washes of 1 min). After sterilization, the seeds were placed in 6-well plates containing 2 mL per well of autoclaved liquid half-strength Murashige & Skoog media (MS, Duchefa Biochemie) supplemented with 1% (w/v) sucrose (pH adjusted to 5.7). Thereafter, seeds were cold stratified by keeping them at 4 °C for 24h, to induce homogenous germination, and grown for three weeks in a 16 h photoperiod (26 μmol m-2 s-1), at 21 °C and 60% relative humidity in a growth chamber (Sanyo, MLR-351).

Tillers obtained from one germinated seedling, as well as *in vitro* propagated tillers, were used for protoplast isolation. The propagated tillers were grown for three weeks in sterile solid MS media supplemented with 3% (w/v) sucrose, thiamine·HCl (1 mg/L), nicotinic acid (0.5 mg/L), pyridoxine (0.5 mg/L), 6-Benzylaminopurine (2 mg/L), and 0.3% Gelrite (all components from Duchefa Biochemie; pH 5.7) and with the same light, temperature, and humidity conditions as described before. Tillers were moved onto fresh media before protoplast isolation. All plates used for germination or propagation were sealed with Micropore™ tape (3M).

### 2.2 Isolation of protoplasts

The effect of the following conditions on the efficiency of protoplast isolation was tested: tissue disintegration instead of cutting, cellulase and mannitol concentrations, enzymatic treatment length, and the use of vacuum infiltration.

For the classical approach, approximately 2 g of fresh weight (gFW) of tillers were cross sectioned into 1–2 mm pieces, placed in a 20 cm Petri dish, and treated with enzyme solution (10 mL per gFW). Since the sectioning of tillers is one of the most time-consuming parts of the protoplast isolation procedure, an alternative method for tiller disintegration was tested as follows.

The same amount of *in vitro* culture derived tillers (2 gFW) was disintegrated using a Waring^®^ laboratory blender (Sigma-Aldrich, catalog number Z272221) in a stainless-steel mini container (250 mL) with 50 mL of sterile mannitol solution (0.5 M), applying blending mode “low” and using short pulses. Four different blending conditions were tested: blending was applied by one, three, five, and ten pulses. Disintegrated tissues were collected by filtering through a 40 µm cell strainer (EASYstrainer™, Greiner Bio-One), moved to a Petri dish with 50 mL of enzyme solution (2% cellulase, composition described in **Table 1**) and incubated for 8 hours. The blending experiments were repeated three times.

**Table 1:** Composition and storage conditions of the solutions used for protoplast isolation and transformation.

Solution	Composition
Enzyme solution^a^	2% cellulase Onozuka R-10, 0.75% Macerozyme R-10, 10 mM MES, 0.6 M mannitol, 20 mM KCl, 10 mM CaCl_2_, 0.1% BSA. pH adjusted to 5.7 with KOH.
W5^b^	2 mM MES, 154 mM NaCl, 125 mM CaCl_2_, 5 mM KCl. pH adjusted to 5.7 with NaOH.
W5A^b^	0.5 mM MES, 5 mM glucose, 154 mM NaCl, 125 mM CaCl_2_, 5 mM KCl. pH adjusted to 5.7 with NaOH.
WI^b^	4 mM MES, 500 mM mannitol, 5 mM KCl. pH adjusted to 5.7 with NaOH.
MMG^b^	4 mM MES, 400 mM mannitol, 15 mM MgCl_2_. pH adjusted to 5.7 with KOH.

a Storage and preparation: Mix MES, KCl, and mannitol, adjust the pH and autoclave. Keep at 4 °C for up to one month. On the day of the experiment, add the enzymes and heat the solution at 55 °C. Add sterile CaCl2 and BSA, when at RT.

b Autoclaved and kept at 4 °C for up to one month.

When sectioning the tillers, different concentrations (w/v) of cellulase (Onozuka R-10, Duchefa Biochemie) were tested: 1.5, 2.0, 2.5 and 3.0%. The complete formulation of the enzyme solution, which included Macerozyme R-10 (0.75% w/v, Duchefa Biochemie), can be found in **Table 1** and is based on previous methods described for the generation of mesophyll protoplasts of perennial ryegrass by Yu et al. (2017) and Davis et al. (2020).

To determine the most suitable digestion length, we incubated the tillers for 8, 12, 16, and 20 h. Four different concentrations of cellulase were tested during these four time points and protoplasts were counted after each of them using fluorescein diacetate (FDA) to determine their viability. 25 µL of FDA solution (5 mg/mL) per mL of protoplast suspension was used, as described by Hu and Huang (2022). Before counting the protoplasts, the suspension treated with enzymes was filtered through a 100 µm cell strainer (EASYstrainer™, Greiner Bio-One) and mounted on a 50 mL tube. The filter was pre-wet with W5 solution (see **Table 1**), the suspension was transferred to the strainer using cut pipette tips (here and always when handling protoplasts) and 4 mL of W5 solution were added to the filter to further increase the number of protoplasts. Then, the suspension was centrifuged for 5 min, 100 g (with minimal acceleration and deceleration) at 11 °C. The supernatant was discarded, and the pellet was resuspended with an equal volume of W5 washing solution (see **Table 1**). To determine the best cellulase concentration and enzymatic treatment duration, the experiments were repeated four times.

Once the best cellulase concentration and enzymatic treatment length were established, different molar concentrations of mannitol (0.2, 0.3, 0.5, and 0.6 M) were tested during a plasmolytic pretreatment of tillers. For each mannitol concentration, 20 mL of mannitol solution was added to a Petri dish with non-sectioned tillers, and the plate was incubated for 1 h in the dark at RT with 75 rpm shaking. Cell viability was determined using FDA as previously described. Finally, we tested the use of vacuum for infiltration of the enzyme solution into the protoplasts (Reed and Bargmann, 2021). For vacuum, 71 kPa of pressure was used for 5 minutes, and the treatment was repeated three times by slowly increasing and releasing the pressure. Cell viability was determined using FDA. The mannitol and vacuum experiments were repeated four times each.

A sucrose gradient was used to further improve the quality and quantity of viable cells. After centrifugation of the cells suspended in W5 solution (as described above), the supernatant was discarded, and the pelleted protoplasts were resuspended in 2 mL of W5A solution (see **Table 1**). In a 15 mL tube, the suspended cells were layered on top of 4 mL of 21% sucrose (w/v) solution and centrifuged for 10 min, 100 g (acceleration and deceleration set to minimum) at 11 °C (Brandt et al., 2020). The layer containing protoplasts after centrifugation was collected and placed into a round bottom tube. 2–3 mL of suspended protoplasts were retrieved. WI solution (see **Table 1**) was added in a 2:1 volume ratio of the collected protoplasts suspension. The tubes were left overnight, or for at least 1 hour, in the dark at 4 °C. Thereafter, the protoplasts were pelleted, and the supernatant was discarded and replaced by half of its volume using WI solution.

### 2.3 Transformation vectors

To evaluate the suitability of the isolated protoplasts for assessing the editing efficiency of CRISPR-Cas9 binary vectors, we designed different gRNAs targeting the first (two gRNAs) and second exon (three gRNAs) of the *LpCBP20* gene, which we used as a testing platform (see **Supplementary files 1**, **2**). The target region was sequenced from cv. Veja and the obtained sequence was used for designing gRNAs with CRISPOR (Concordet and Haeussler, 2018). The latest available *L. perenne* genome assembly was used as a query for the specificity and off-target calculations (GCF_019359855.1) (Frei et al., 2021). gRNAs with high specificity to the targeted region (score equal to or greater than 85) and low prediction of off-targets (below 10 predicted off-targets and 0 off-targets close to a PAM region), were selected.

Two different plasmids were tested: pHSE401/EGFP and pTRANS_HiGRFdGm1. Both plasmids contain fluorescent markers, EGFP and ZsGreen, respectively. The plasmid pHSE401 was a gift from Qi-Jun Chen (Addgene plasmid # 62201) and has a single gRNA cassette (Xing et al., 2014) (**Supplementary file 3**). Two versions of the plasmid containing a different gRNA (gRNA 196 or gRNA 229) were created following the Golden Gate assembly described in Xing et al. (2014), targeting the second exon of the *LpCBP20* gene. The resultant vectors were named p196 and p229.

Plasmid, pTRANS_HiGRFdGm1, can fit five different guides separated by tRNA repeats. This plasmid was a kind gift from Dr. Sergei Kushnir (Teagasc) and based on vector pTRANS_210d (Addgene plasmid # 91109) described in Čermák et al. (2017), which was used as a backbone. The backbone contains different expression cassettes: one cassette coding for ZsGreen, another cassette coding for a morphogenic regulator (data not published), and a cassette containing an intronic HPTII CDS to provide hygromycin resistance to the transformed plant material (map of the final transformation vector available in **Supplementary file 4**).

The two guide RNAs previously mentioned (g196 and g229) were used in combination with two others that targeted the first exon (g9 and g22) of the *LpCBP20* gene and an additional guide targeting the second exon of the gene (g220). This vector was labeled as pCBP20_5g (see **Supplementary file 4**) and was assembled by Dr. Anete Borodušķe (University of Latvia) using the Golden Gate approach into pTRANS_HiGRFdGm1 backbone, following the cloning protocol described by Čermák et al. (2017). The plasmid pMOD_A1110 (Addgene plasmid # 91031) was used as module A and encoded a wheat codon optimized Cas9 nuclease. Plasmid pMOD_B2303 (Addgene plasmid # 91068) was used as module B and encoded a polycistronic cassette suitable for the expression of multiple gRNAs. Plasmid pMOD_C0000 (Addgene plasmid # 91081) was used as module C providing the necessary bases needed for the final assembly of the transformation vector using Golden Gate cloning.

In addition, another *L. perenne* gene, *LpCRPK1*, was targeted using a different plasmid. This plasmid was based on the Level 2 vector EC67907 kindly provided by Wendy Harwood & Cristobal Uauy (Addgene plasmid # 211794) (Lawrenson et al., 2024). The same ZsGreen cassette present in plasmid pTRANS_HiGRFdGm1 was inserted into the Level 2 vector to create the plasmid piCas9_ZsGreen (see **Supplementary file 5**). This plasmid was used to generate a transformation vector encoding 6 gRNAs targeting three different paralogs of the *LpCRPK1* gene (more information present in **Supplementary files 1**, **2**). The assembly was done according to Lawrenson and colleagues (Lawrenson et al., 2024). Six different Level 1 vectors were used, each one encoding a different gRNA. Plasmid EC70188 (Addgene plasmid # 209449) encoded gRNA 190-1, plasmid EC70196 (Addgene plasmid # 209457) encoded gRNA 190-2, plasmid EC70204 (Addgene plasmid # 209465) encoded gRNA 234-1, plasmid EC70191 (Addgene plasmid # 209452) encoded gRNA 234-2, plasmid EC70199 (Addgene plasmid # 209460) encoded gRNA 232–1 and plasmid EC70207 (Addgene plasmid # 209468) encoded gRNA 232-2. Plasmids pAGM8031 (Addgene plasmid # 48037) and pAGM8079 (Addgene plasmid # 48041) were used as Level M accepters, and plasmids PICH50900 (Addgene plasmid # 48047) and PICH50927 (Addgene plasmid # 48049) were used as Level M linkers. Plasmid piCas9_ZsGreen was used as the Level 2 accepter and plasmid PICH41822 (Addgene plasmid # 48021) was the Level 2 linker.

Two gRNAs were designed for each *LpCRPK1* paralog, targeting the first and second exons. The sequences of each paralog were amplified and sequenced from a perennial ryegrass freezing sensitive genotype (more information available in Pashapu et al. (2024) and used for gRNA design with CRISPOR.

To further estimate the transformation efficiency, empty vectors (i.e., not coding for gRNAs) were used as controls and designated as pEGFP and pDelta for the single gRNA and multi gRNA plasmid, respectively, in comparison to the vectors used to target the *LpCBP20* gene. For the plasmid containing six gRNAs targeting different paralogs of the *LpCRPK1* gene, an empty vector not coding gRNAs was labeled as pCtrl_iCas9 and used as a control.

The sequences of the primers and gRNAs used in this study can be found in **Supplementary files 2**, **6**. All the amplifications performed in this study were done using ThermoFisher’s Phusion™ High-Fidelity DNA Polymerase to reduce the chance of polymerase-induced nucleotide variations, as recommended by Tsakirpaloglou et al. (2023). All the transformation vectors, with and without gRNAs, were checked for correct assembly using an external whole plasmid sequencing service.

### 2.4 PEG-mediated protoplasts transformation

For the transformation procedure, the volume of protoplasts suspension in MMG (see **Table 1**) was adjusted to contain 3 × 10^5^ cells. To change the solution from WI, in which they were placed after performing the sucrose cushion, the needed volume for the previously mentioned cell density was placed in a 2 mL tube, which was centrifuged at 100 g (always with acceleration and deceleration set at minimum), RT for 5 min. The supernatant was discarded and replaced by the same volume of MMG solution. Tubes containing 10 µg of either control or gRNA encoding vectors were prepared. The plasmid DNA was obtained by midi-prepping *E. coli* encoding said plasmids using the PureLink™ HiPure Plasmid Midiprep Kit (Invitrogen™) following the instructions described by the manufacturer. The protoplasts suspended in MMG were added to the tubes containing the plasmids and mixed gently. Freshly prepared PEG solution (0.4 g/mL PEG 4000, 0.2 M mannitol, 0.1 M CaCl_2_) was added to a volume ratio of 1:1 and the suspension was incubated in the dark for 15–20 min, leaving the tubes horizontally after gentle mixing. Following incubation, a wash was performed with W5 solution. The tube was then centrifuged at 100 g and RT for 5 min. The supernatant was discarded, and the pellet was resuspended in 1 mL of WI. The suspension was then passed into a 24-well plate, the wells of which were precoated with 5% BSA. The plate was incubated for 48 h in the dark. This transformation method was adapted from (Brandt et al., 2020). After two days of incubation, samples were collected to calculate the percentage of fluorescent protoplasts and determine the transformation efficiency using an inverted fluorescence microscope (Zeiss Axiovert 200M) and a Neubaer chamber (BLAUBRAND). Genomic DNA was extracted from transformed cells according to Weigel and Glazebrook’s protocol with some modifications (Weigel and Glazebrook, 2009). DNA from non-transformed protoplasts was extracted following the same method and used for comparisons.

### 2.5 Determination of editing efficiency

Genomic DNA from transformed and non-transformed samples was used to perform PCR amplification of the targeted genes *LpCBP20* and *LpCRPK1*. These amplifications were done using ThermoFisher’s Phusion™ High-Fidelity DNA Polymerase. To ensure the reliability of the results, the genomic DNA of untransformed protoplasts belonged to the same suspension of cells used for the different transformations.

The generated amplicons were sequenced by Sanger, and the obtained trace data was used to determine the presence of indels in the transformed samples. The editing efficiency of the gRNAs was established using the TIDE tool. Based on what was recommended by the authors of TIDE, the cut-off for the obtained data was: R^2^ equal to or greater than 0.9 and indel frequencies considered as statistically significant by TIDE (P < 0.001) (Brinkman et al., 2014).

### 2.6 Statistical analysis and graphical representations

The statistical analysis and graphs were done using GraphPad Prism 10.2.0 for Windows (GraphPad Software, Boston, Massachusetts USA, www.graphpad.com). A two-way ANOVA test with the Geisser-Greenhouse correction was used to detect statistically significant variations between the different enzymatic treatment time points and cellulase concentrations. Tukey’s multiple comparison was used to determine the specific cellulase concentration and enzymatic treatment length that produced the significantly highest number of alive cells. For the blending experiments, pairwise comparisons were assessed by Tukey’s multiple comparisons test to analyze the different blending intensities. To evaluate the number of alive cells produced by the different mannitol pretreatments, a one-way ANOVA analysis together with the Geisser-Greenhouse correction was used. To determine the mannitol concentration that yielded the significantly highest number of alive protoplasts, Tukey’s multiple comparison was used. A two-tailed unpaired t-test was used to calculate statistical significance for samples with and without vacuum infiltration. For the analysis of the transformation efficiency of the pHSE401/EGFP plasmid, a Welch and Brown-Forsythe ANOVA test was performed together with Dunnett’s multiple comparison test. To evaluate the transformation efficiency of the pTRANS_HiGRFdGm1 plasmid coding (pCBP20_5g) and not coding for a gRNA (pDelta), an unpaired t-test was performed. A similar evaluation was done to analyze the transformation efficiency of the plasmid targeting *LpCRPK1* paralogs, by comparing it with the transformation data of a plasmid not encoding gRNAs (pCtrl_iCas9). The editing efficiency of the plasmid with one guide RNA (p196 and p229) was evaluated using an unpaired t-test with Welch’s correction. For the vector encoding 5 different guides (pCBP20_5g), the efficiency of the gRNAs was analyzed using a Welch and Brown-Forsythe ANOVA test together with Dunnett’s multiple comparison test. The same statistical analysis was used to evaluate the efficiency of the gRNAs part of the plasmid targeting different paralogs of *LpCRPK1* (piCas9_CRPK1).

## 3 Results

### 3.1 Protoplast isolation

**Figure 1:**
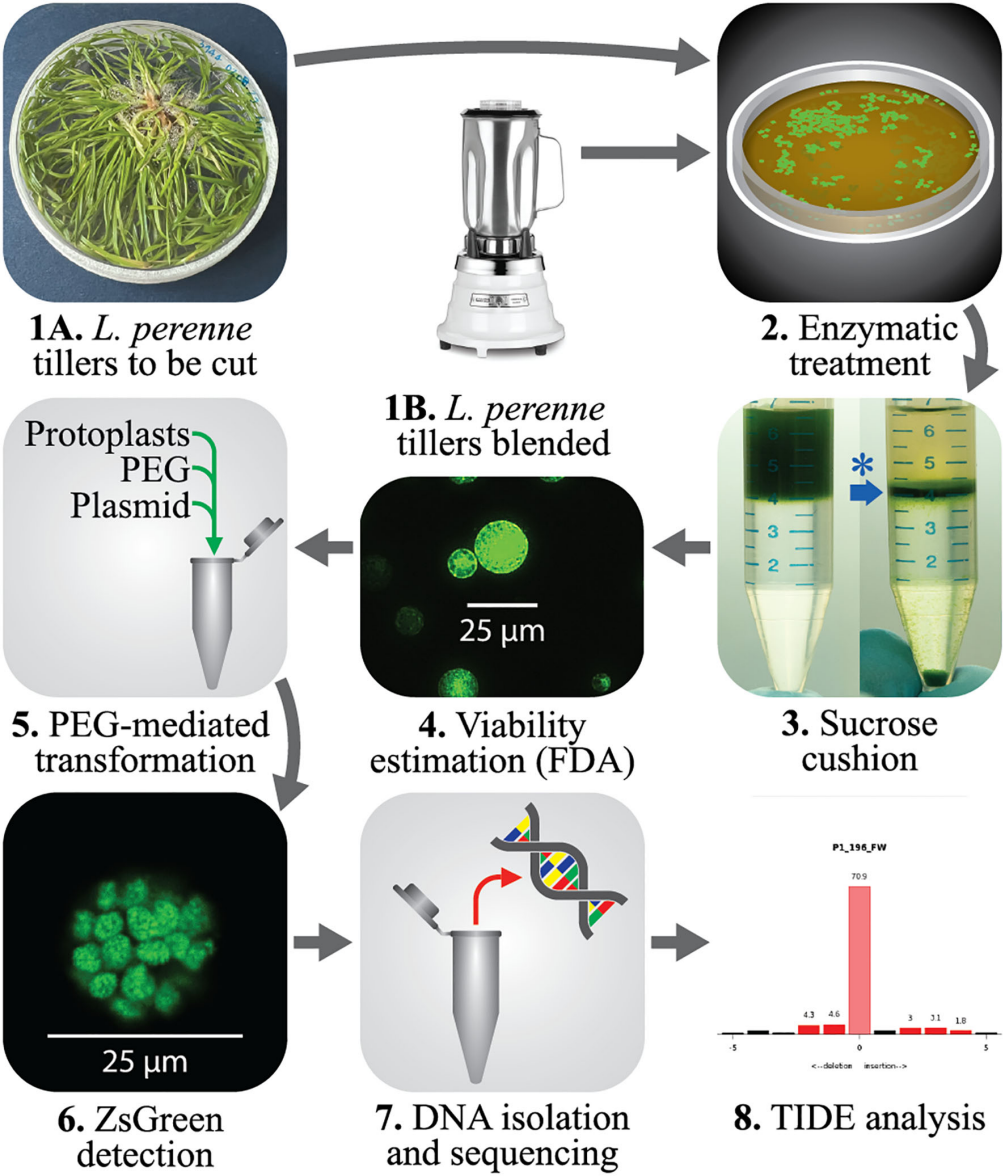
Schematic representation of the methods used for isolating perennial ryegrass protoplasts and transforming them for the screening of gRNAs. **1A.***L. perenne in vitro* grown tillers or seedlings were used as starting material and processed using a razor blade. **1B.** Alternatively, the tillers or seedlings of perennial ryegrass were disintegrated using a Warning laboratory blender. **2.** The tillers were treated with an enzyme solution containing 2% cellulase for 8 h in dark conditions. **3.** A suspension of alive protoplasts was obtained (asterisk and blue arrow) using a sucrose cushion (21%). **4.** The protoplasts suspension was kept overnight at 4 °C, and the cell density and viability were estimated using FDA staining. **5.** 3 × 10^5^ protoplasts were used for PEG-mediated transformation of plasmids encoding 1 or multiple gRNAs. **6.** After 48 h of incubation, the transformation efficiency was calculated by observing green fluorescence in the protoplasts. **7.** DNA from transformed and non-transformed samples was isolated and used for Sanger sequencing. **8.** The presence of indels in the genome of transformed protoplasts was analyzed with TIDE. Image created with Adobe Illustrator 2023.

In this study, we tested two methods for isolation of protoplasts from perennial ryegrass. One method followed the classical cutting of the tillers in small pieces before the enzymatic treatment, while the other was a novel way of disintegrating the plant tissue using a blender. In both cases, the amount of initial plant tissue was 2 gFW. The experimental design is shown in **Figure 1**, describing the workflow from collecting the tissue till the outcome of the transformation of the obtained protoplasts.

**Figure 2:**
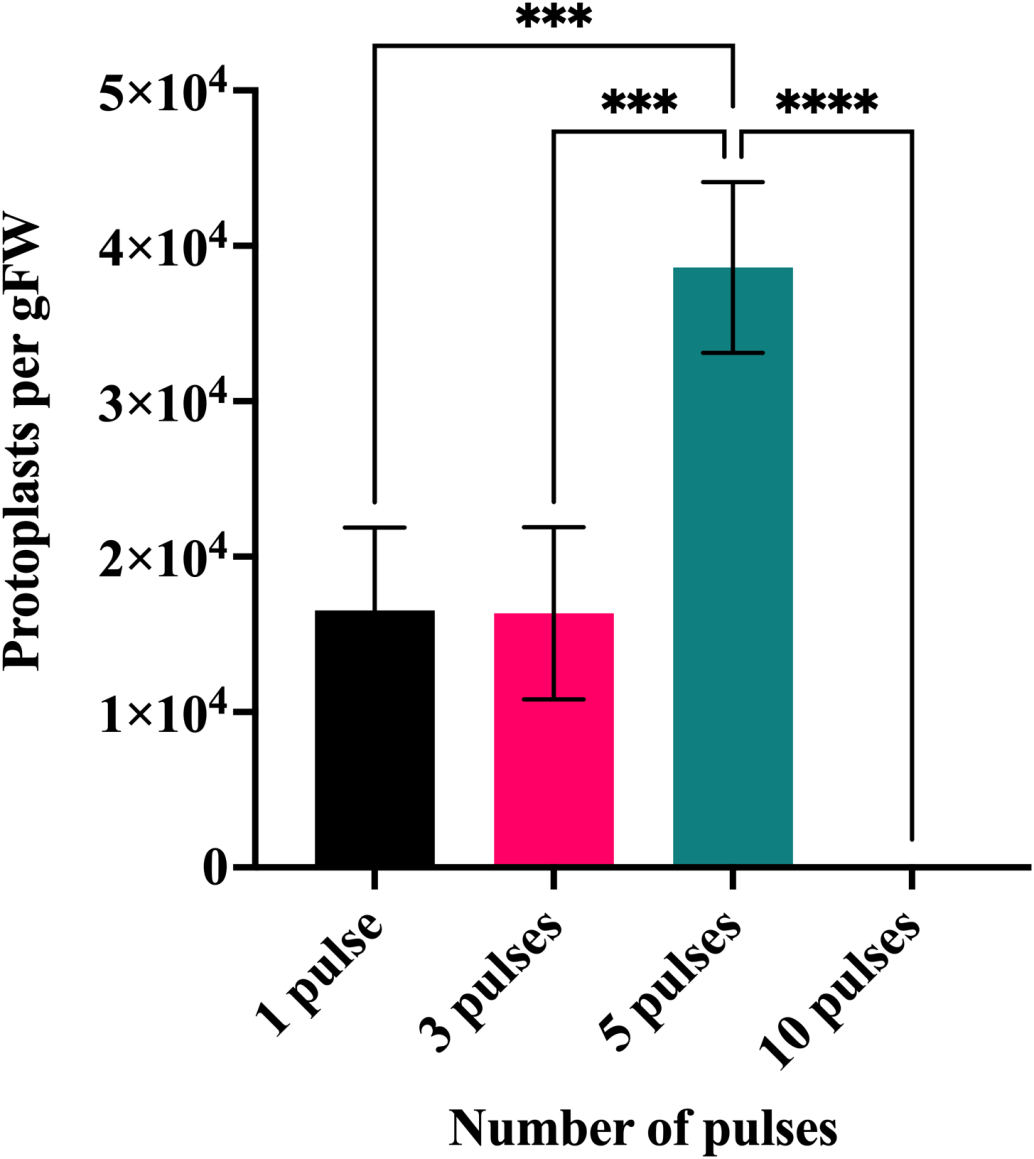
The effect of tissue disintegration on the efficiency of protoplast isolation. *In vitro* shoot culture derived from *L. perenne* tillers were disintegrated by blending before enzymatic treatment. Four different blending regimes were compared – disintegration with a single short blending pulse, three pulses, five pulses, and ten pulses. Each bar represents the average number of viable protoplasts per g of fresh weight (gFW) determined by FDA staining of three biological replicates; error bars represent standard deviation. ***P ≤ 0.001 and ****P ≤ 0.0001.

The yield of isolated protoplasts generated when blending the tillers instead of cutting them can be seen in **Figure 2**. Using 5 pulses provided a statistically significant higher number of alive cells on average (3.86 × 10^4^ cells per gFW) when compared with the use of 1, 3 or 10 pulses.

**Figure 3:**
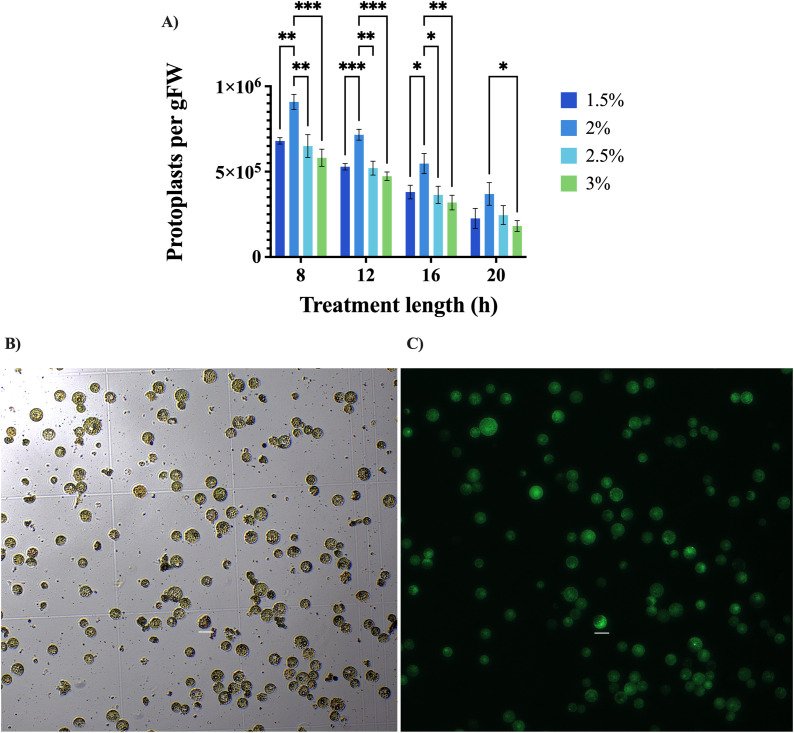
Isolation of protoplasts using the classical method. **(A)** Comparison of four enzyme solutions and four different enzymatic treatment lengths. Bars plot portrays results for the different cellulase concentrations used at each time point. The Y-axis shows the number of counted alive cells, using FDA and a hemocytometer, as protoplasts per g of fresh weight (gFW). The X-axis displays the duration of the enzymatic treatment in hours. Error bars represent standard deviation. *P ≤ 0.05, **P ≤ 0.01 and ***P ≤ 0.001. **(B)** Isolated cells under bright field conditions. **(C)** Viable protoplasts showing fluorescence due to FDA conversion into fluorescein. Scale bar represents 20 µm.

**Figure 4:**
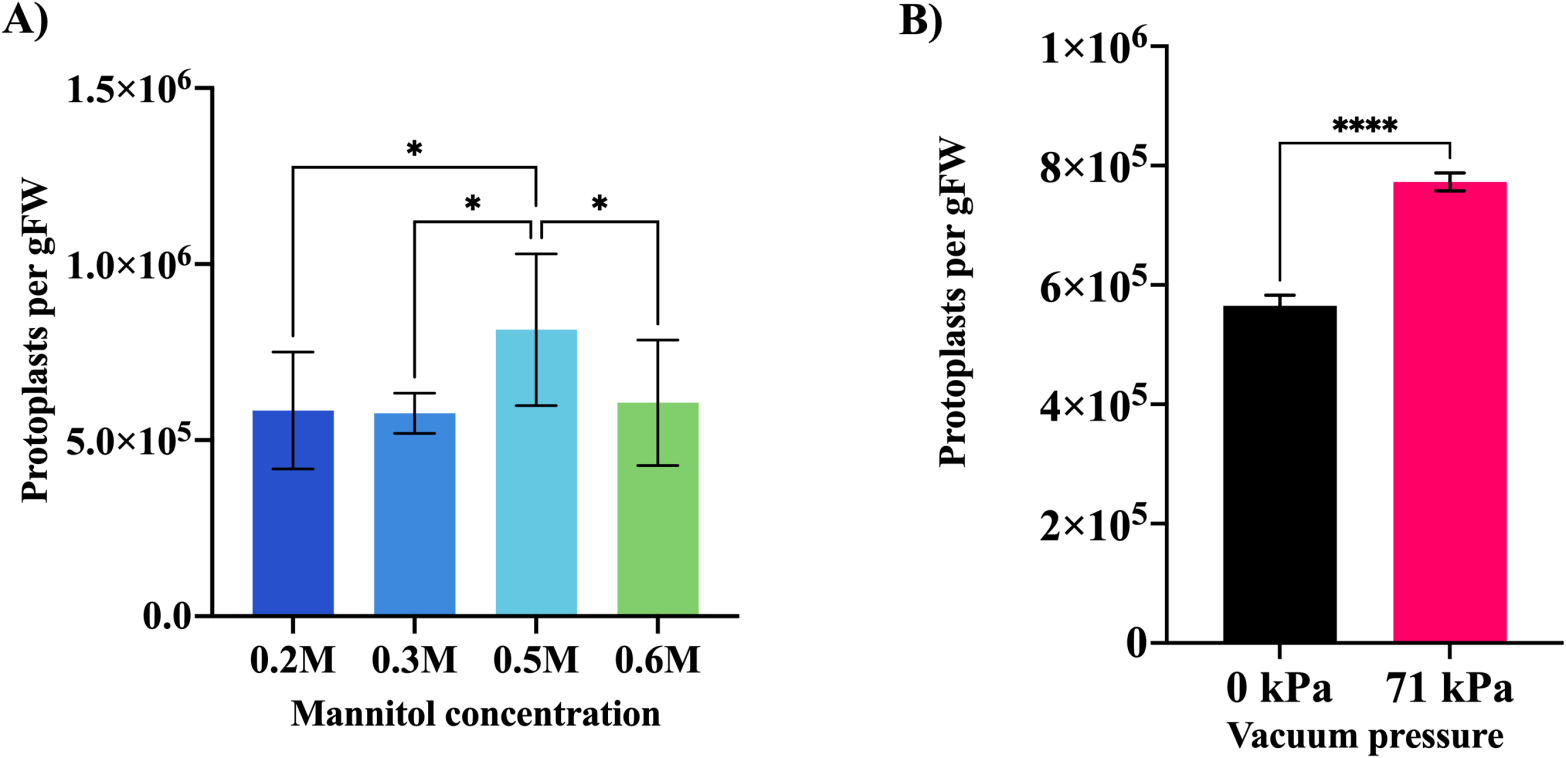
Mannitol pretreatment and vacuum infiltration experiments. **(A)** Graph showing the comparison between four mannitol concentrations used for the pretreatment of intact tillers. The Y-axis shows the number of counted alive protoplasts using FDA and a hemocytometer (protoplasts per g of fresh weight, gFW). The X-axis shows the molar concentration of mannitol used per treatment. **(B)** Comparison between the use of 71 kPa of vacuum and the absence of pressure. The Y-axis shows the number of counted alive protoplasts using FDA and a hemocytometer (protoplasts per gFW). The X-axis shows the magnitude of vacuum pressure used in kPa. Each experiment was repeated four times. Error bars represent standard deviation. *P ≤ 0.05 and ****P ≤ 0.0001.

In the case of the classical method, cutting tillers with a razor blade, four different incubation times (8, 12, 16, and 20 h) were used to test four different cellulase concentrations (1.5, 2, 2.5, and 3%) present in the enzyme solution. **Figure 3** shows the number of counted alive protoplasts after testing these two parameters. The use of 2% cellulase gave the best results in terms of alive cells, independently of the enzymatic incubation time. 8 h incubation produced significantly better results than all other digestion lengths, since after this period the number of protoplasts started to decrease (**Figure 3**). Therefore, the best yield in terms of alive protoplasts per gFW, approximately 9 × 10^5^, was obtained using an enzyme solution with 2% cellulase to perform the enzymatic digestion for 8 h. The use of a solution with 0.5 M mannitol for pretreatment provided a significantly higher yield, approximately 8 × 10^5^ protoplasts per gFW, than the other mannitol molar concentrations (**Figure 4A**). Finally, the application of 71 kPa of vacuum pressure gave significantly better results than when no vacuum was used, yielding around 7.7 × 10^5^ alive cells per gFW (**Figure 4B**). It must be noted though, that even when vacuum was not used, around 6 × 10^5^ living cells per gFW were counted. Increasing vacuum pressure improved protoplast yields also for the blender method, although the difference was not statistically significant (data not shown).

### 3.2 Protoplast transformation

**Figure 5:**
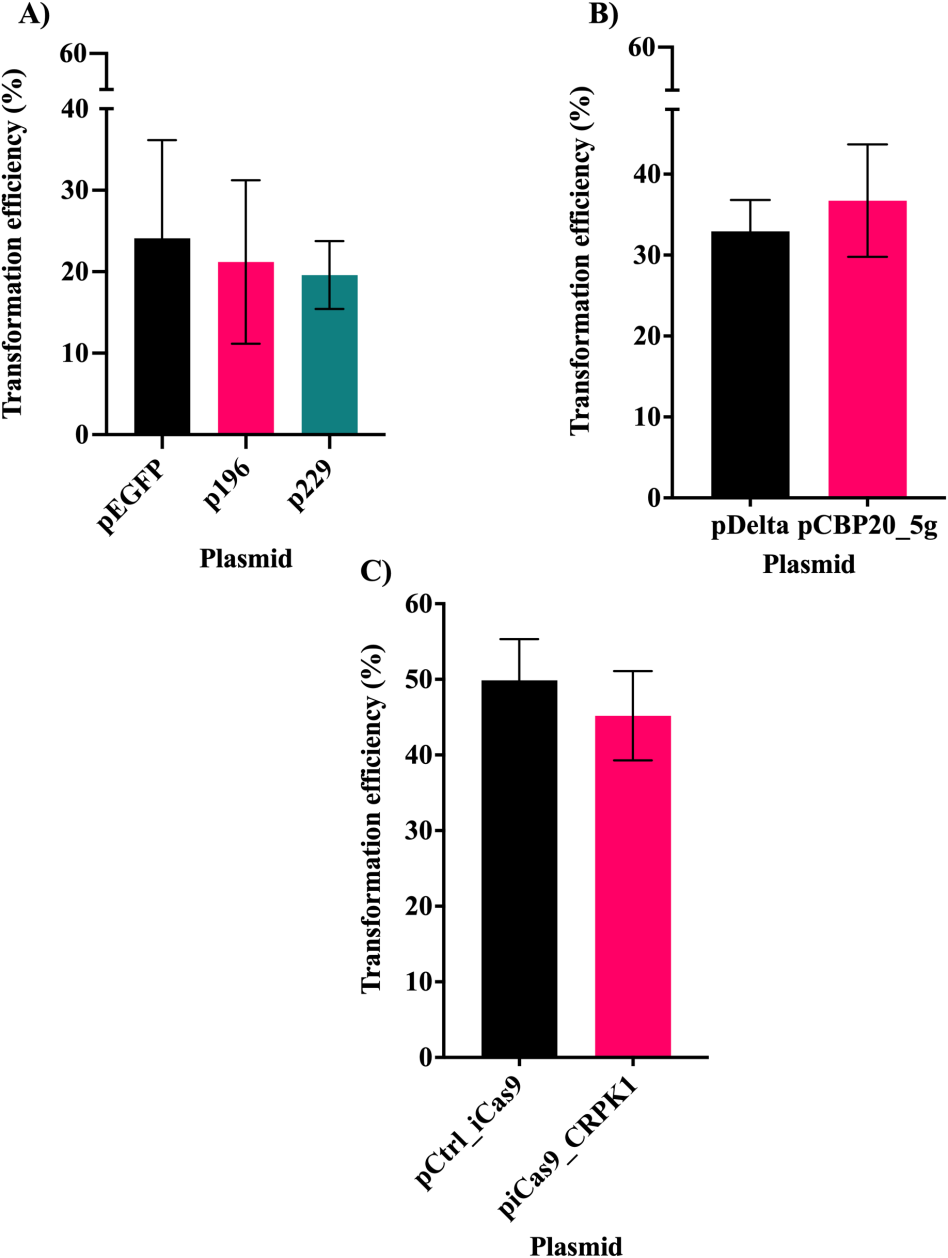
Transformation efficiency of the vectors used to transform *L. perenne* protoplasts. **(A)** Transformation efficiency observed when using vectors derived from the pHSE401/EGFP plasmid. pEGFP: control plasmid not coding for any gRNA; p196 coding for gRNA 196 and p229 for gRNA 229. The transformation using the pHSE401/EGFP vectors was repeated 6 times. **(B)** Transformation efficiency observed when using vectors derived from the pTRANS_HiGRFdGm1 plasmid. pDelta: control plasmid not coding for any gRNA; pCBP20_5g coding for five gRNAs. The transformation using the pTRANS_HiGRFdGm1vectors was repeated 12 times. **(C)** Transformation efficiency of the plasmids encoding an intronized Cas9. pCtrl_iCas9: control plasmid not encoding gRNAs; piCas9_CRPK1 coding for six gRNAs. The transformation using the control plasmid was repeated 4 times and 12 times in the case of the vector encoding six gRNAs. The Y-axes show the transformation efficiency derived from the percentage of fluorescent cells observed. Error bars represent standard deviation. No statistically significant differences were observed.

Fluorescence was detected by microscopy in the protoplasts transformed with both types of plasmids, targeting *LpCBP20* or *LpCRPK1*. This indicated a positive transformation of the cells using PEG. The average number of fluorescent protoplasts with respect to the total counted cells for each of the transformation vectors can be seen in **Figure 5**. In the case of the single gRNA vectors, no statistically significant differences were observed between the control plasmid (pEGFP) and the plasmids encoding one gRNA (p196and p229 (**Figure 5A**). The other vectors targeting *LpCBP20* also presented a not statistically significant difference (**Figure 5B**). Since ZsGreen has proved to be brighter than EGFP (Susič et al., 2014; Cho et al., 2019), the transformation efficiency of the two types of vectors used to edit *LpCBP20* cannot be compared. Moreover, for the plasmids encoding ZsGreen and an intronized Cas9 no statistically significant differences were observed between the control plasmid (pCtrl_iCas9) and the vector encoding 6 gRNAs (piCas9_CRPK1) (**Figure 5C**). Although vectors encoding an intronized Cas9 appeared to have a higher transformation efficiency than those containing the ZsGreen cassette and a non-intronized nuclease, this difference was not statistically significant.

### 3.3 Editing efficiency

DNA was extracted from the protoplasts after transformation and Sanger-sequenced. Representative figures showing the amplified PCR products can be seen in **Supplementary file 7**.

**Figure 6:**
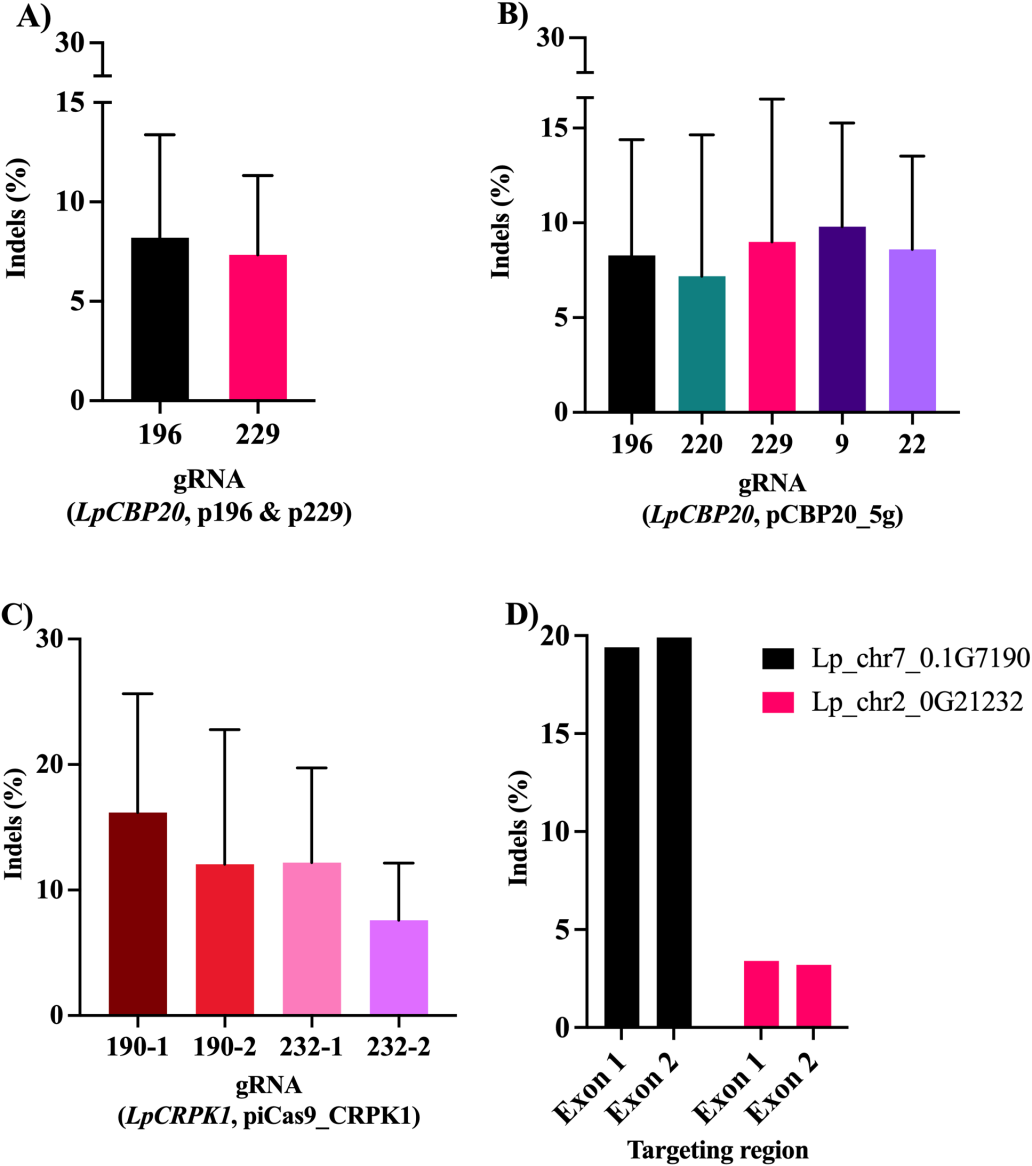
Editing efficiency of the different vectors used to transform *L. perenne* protoplasts calculated as frequency of indels with TIDE. **(A)** Editing efficiency of the gRNAs targeting the second exon of gene *LpCBP20* when transforming protoplasts with pHSE401/EGFP-derived plasmids encoding gRNA 196 or gRNA 229. **(B)** Editing efficiency of the five gRNAs targeting *LpCBP20* gene when multiplexed into the pCBP20_5g vector. **(C)** Editing efficiency of gRNAs encoded in the vector piCas9_CRPK1. **(D)** Indels frequencies of the transformation event in which both targeted exons of two of the *LpCRPK1* paralogs (*LpCRPK1–190* and *LpCRPK1-232*) were edited. The data plotted corresponds to results when the cut-offs in TIDE were P < 0.001 (for each transformation event) and R² ≥ 0.9. After filtering, the repetitions considered for pHSE401/EGFP-derived plasmids were as follows: n = 5 for 196 and n = 3 for 229. For the pCBP20_5g plasmid repetitions were n = 3 for gRNAs 9 and 22; n = 4 for gRNAs 196 and 229; n = 5 for gRNA 220. For the piCas9_CRPK1 plasmid the repetitions were n = 11 for gRNA 190-1; n = 13 for gRNA 190-2; n = 8 for gRNA 232-1; n = 3 for gRNA 232-2. Error bars represent standard deviation. No statistically significant differences were observed.

Decomposition-based analysis with the TIDE program was performed and this resulted in the detection of the frequency of indels in the pool of the protoplasts transformed with plasmids coding for one or multiple gRNAs. These results are shown in **Figures 6A–C** and depict only the events that passed the threshold set for TIDE: indel frequencies with a R^2^ equal to or greater than 0.9 and P < 0.001. For the plasmids targeting *LpCBP20*, the average editing efficiencies were between 5 and 10%. While no statistically significant difference was observed between the editing efficiency of the plasmids targeting *LpCBP20*, it seemed that in our experiments the plasmids coding for a single gRNA presented on average slightly lower frequencies of indels (7.8%, **Figure 6A**) than the multiplex plasmid (8.6%, **Figure 6B**). In the case of the plasmid targeting *LpCRPK1*, the editing efficiency of all the gRNAs was above 10%, except for gRNA 232-2 (**Figure 6C**). Only samples that met the previously described TIDE threshold for efficiency were plotted and analyzed. Due to this cutoff, the results corresponding to gRNAs 234–1 and 234–2 were excluded from further analysis. No statistically significant differences were observed between the different gRNAs encoded in the vector targeting *LpCRPK1*. Additionally, in one of the analyzed samples two of the targeted paralogs showed the presence of indels in both exons (**Figure 6D**). While this result belongs to only one sample, it is a good representation of the possibility of using the piCas9_CRPK1 to simultaneously edit multiple genes. Furthermore, it proves the reliability of our method when analyzing multiplex genome editing reagents.

Importantly, the genome editing efficiency in all cases was enough for proceeding with plant transformation using those vectors. A representation of the indels predicted by TIDE for the two multiplex transformation vectors used in this study can be found in **Supplementary file 8**.

## 4 Discussion

Developing reliable genome editing protocols for *Lolium perenne*, an important forage grass, remains challenging due to the limited efficiency of calli transformation. To overcome this, the use of protoplasts has emerged as valuable intermediate step for screening genome editing reagents *in vivo*, such as plasmids and gRNAs. This approach has been successfully used in other grass species like rice, wheat and maize (Shan et al., 2013; Brandt et al., 2020; Fierlej et al., 2022). Here, we describe the development and validation of two approaches for isolating enough perennial ryegrass protoplasts suitable for transformation assays. The PEG-mediated transformation of protoplasts served as a platform to evaluate, *in vivo*, the editing efficiency of three different types of gRNA encoding vectors.

Standard protoplast isolation techniques are based on the enzymatic degradation of cell walls, in combination with gentle mechanical force agitation. In grasses, the most frequent starting material for protoplasts isolation are leaves or tillers. These are normally processed before enzymatic treatment, often by cutting them with a razor blade, to favor enzyme activity. This manual cutting is one of the most labor-intensive and time-consuming parts of most protoplast isolation methods. As a novel alternative for processing tillers of *L. perenne*, we tested the use of a blender to simplify and speed up this process. While this approach decreased the yield of obtained protoplasts by approximately one order of magnitude compared to the razor cutting method, it significantly decreased handling time. The choice between these methods should be based on the downstream applications of the isolated cells. Researchers should consider whether maximizing yield or minimizing labor and processing time is the most important factor for their needs.

The mix of enzymes used for protoplasts isolation varies among different plant species due to differences in cell wall composition (Chawla, 2009; Davey et al., 2010). To optimize this step for *L. perenne*, we tested four different cellulase concentrations: 1.5, 2, 2.5, and 3%. This was supported by previous evidence indicating that cellulose is the main component of *L. perenne* cell walls (approx. 46%) (Gordon et al., 1985), and by informatic models showing an important role for cellulase in degrading perennial ryegrass mesophyll cell walls (Vetharaniam et al., 2014). The rest of the enzymatic solution components were maintained, as previously reported by Yu et al. (2017) and Davis et al. (2020). The enzymatic treatment duration also depends on the plant species, and that is why we tested the effect of different incubation periods. Previously published protocols for *L. perenne* range from 6 h (Yu et al., 2017) to 20 h (Davis et al., 2020) of enzymatic treatment. Based on this variability, we evaluated four different incubation durations: 8, 12, 16 and 20 h. Among the tested combinations, the best performing conditions were 2% cellulase concentration and 8h of enzymatic treatment.

Another important part of the isolation procedure is the use of a pretreatment before the plant material is exposed to the enzyme solution. These pretreatments can be done before or after the tillers are processed or cut and are meant to further improve the number of isolated alive cells since non-ionic solutes have been shown to induce the separation of cell membranes from cell walls (Reed and Bargmann, 2021). We tested a range of mannitol concentrations before processing the tillers or seedlings and found that a 0.5 M solution generated the highest number of alive protoplasts. In addition, we also evaluated the use of vacuum infiltration to enhance enzyme penetration into the plant material. This led to an increase in the protoplast yield. Contrary to previous perennial ryegrass protocols (Yu et al., 2017; Davis et al., 2020), we introduced an additional step consisting of using a sucrose cushion to reduce cellular debris. This helped minimize interference during transformation and downstream use of the protoplasts (Chen et al., 2023).

In line with studies in other plant species, we present a protoplast-based platform for testing the activities of gRNAs prior to their use in non-transient transformations (Brandt et al., 2020; Fierlej et al., 2022; Lee et al., 2023). Although Zhang and colleagues (Zhang et al., 2020) mention the use of perennial ryegrass protoplasts for testing gRNAs, their brief communication does not include data on transformation or editing efficiency. To our knowledge, this is the only study reporting genome editing of *L. perenne* protoplasts. Two other publications presented the transformation of perennial ryegrass protoplasts in a general manner, without focusing on editing purposes (Yu et al., 2017; Davis et al., 2020).

In all these previous studies, PEG was used for the transformation of protoplasts. This method is widely adopted across plant species due to its simplicity and efficiency. Additionally, it does not require specific equipment like electroporation-based transformations. In our study, PEG-mediated transformation was also effective. Fluorescent signal from EGFP or ZsGreen confirmed the successful delivery of the tested reagents.

Fluorescence was detected in over 10% of protoplasts transformed with vectors encoding single gRNAs (p196 and p229), more than 30% of cells transformed with the plasmid encoding 5 gRNAs (pCBP20_5g), and over 40% of protoplasts transformed with the vector targeting *LpCRPK1* (piCas9_CRPK1). These results suggest that the multiplexed vectors may achieve higher transformation efficiency in comparison to those encoding a single gRNA. However, a direct comparison cannot be drawn between the vectors used to edit *LpCBP20*, since they encode two different fluorescent proteins. Nonetheless, there is a possibility that the EGFP expression is weaker than usual because of transcriptional silencing. In the pHSE401/EGFP plasmid there are two other transcriptional units under the control of CaMV 35S promoters, in addition to the EGFP cassette (Altpeter et al., 2016; Anjanappa and Gruissem, 2021).This could explain why the vectors encoding a single gRNA presented lower transformation efficiencies than the other types of plasmids.

While no statistically significant differences were detected among the multiplex plasmids, the higher number of fluorescent cells observed when using the intronized nuclease vector may be attributed to improved experimental handling at later stages of the study.

The DNA delivery method used in this study generated enough transformed protoplasts for testing suitable gRNAs. We assessed the editing efficiency of different guides by Sanger sequencing of genomic DNA followed by TIDE decomposition analysis. This approach provides an estimation of the frequency of indels, induced by a Cas nuclease guided by a specific gRNA, present in a mixed population of cells (Bennett et al., 2020; Brockman et al., 2023; Aoki et al., 2024). TIDE has been reported as particularly well-suited for detecting low-frequency editing events, especially with frequencies below 10%, which was relevant in several of our samples (Brockman et al., 2023; Aoki et al., 2024). In our study, indels were detected in most cases, even though in some samples the results showed low R^2^ values, indicating a weaker fit with the statistical model. When comparing the editing efficiency of the vectors targeting the *LpCBP20* gene, no statistically significant differences were observed between the single and multiple gRNA coding vectors. Despite this, the overall performance of both vector types supports their use in downstream applications.

The vector encoding six gRNAs targeting three different paralogs of *LpCRPK1*, achieved editing efficiencies above 10% for three of the gRNAs (190-1, 190–2 and 232-1) and close to 8% in the case of gRNA 232-2. The relatively higher editing rates observed for most of these gRNAs may be attributed to the presence of an intronized Cas9 nuclease in the transformation vector. Previous reports have shown that the addition of introns in the Cas9 coding sequence can enhance gene expression by promoting higher transcription and translation levels, a phenomenon known as intron-mediated enhancement, which can lead to improved editing efficiencies (Rose et al., 2011; Castel et al., 2019; Grützner et al., 2021). The intronized Cas9 used in our study contains thirteen introns and has previously shown editing levels exceeding 90% in barley (Lawrenson et al., 2024). In one of the analyzed samples, two of the targeted paralogs presented indels in both exons, further indicating multiplex editing.

## 5 Conclusions

From the two different approaches developed and tested for processing perennial ryegrass material before their enzymatic treatment, using a blender was faster and easier but resulted in a lower number of alive cells than the more classical approach based on sectioning the material with a scalpel Additionally, the best composition of an enzymatic solution together with the best performing incubation time were determined. The inclusion of a sucrose gradient, not previously reported in the isolation of perennial ryegrass protoplasts, further increased the isolation of viable cells. Similar to what has been proven in other grasses, the method described in our study showed that perennial ryegrass protoplasts are suitable for testing *in vivo* the outcome of binary vectors used for gene editing. This evaluation allows screening suitable vectors or gRNAs before their application in the transformation of *L. perenne* material, such as calli. The *in vivo* selection of gRNAs can speed up the generation of mutant plants with interesting phenotypes, such as stress-related tolerance, especially in non-model organisms or species recalcitrant to transformation and regeneration. The proposed method can be used to test other editing reagents, such as RNPs, which could allow the generation of transgene-free mutant plants. As the climate is changing faster than plants can adapt to it, having the ability to generate new genotypes with improved stress tolerance is paramount for sustainable food and feed production.

## Data Availability

The original contributions presented in the study are included in the article/**Supplementary Material**. Further inquiries can be directed to the corresponding author.

## Supplementary material

Supplementary file 1 **(a)** Graphic representation of the first and second exon of gene *LpCBP20* together with the gRNAs and primers used in this study. (**b**) Graphic representation of the first and second exons of the *LpCRPK1* paralogs targeted in conjunction with the gRNAs and primers used.

Supplementary file 2 Sequences of gene *LpCBP20* and *LpCRPK1* paralogs containing location of primers, gRNAs and targeted exons.

Supplementary file 3 Map of the pHSE401/EGFP plasmid.

Supplementary file 4 (**a)** Map of the transformation vector pCBP20_5g. (**b**) Graphical representation of the transcriptional unit encoding the five gRNAs in the pCBP20_5g plasmid.

Supplementary file 5 (**a)** Map of the transformation vector piCas9_CRPK1. (**b**) Graphical representation of the transcriptional unit encoding the six gRNAs present in piCas9_CRPK1.

Supplementary file 6 **(a)** Table containing the primers used in this study. (**b)** Table containing the gRNAs used in this study.

Supplementary file 7 Images showing the amplification of the PCR products used for sequencing and subsequent analysis for indel assessment with TIDE.

Supplementary file 8 (**a)** Representation of the indel frequencies of one editing event using the pCBP20_5g transformation vector. (**b)** Representation of the indel frequencies of one editing event when targeting paralogs *LpCRPK1–190* and *LpCRPK1–232* using the piCas9_CRPK1 transformation vector.
