# Supplementary material for: Isolation and transformation of perennial ryegrass (Lolium perenne L.) protoplasts for the in vivo assessment of guide RNAs editing efficiency
Source: Front Plant Sci. 2026 Jan 16;16:1744085. doi: 10.3389/fpls.2025.1744085 (PMC12856575; doi:10.3389/fpls.2025.1744085)
Supplement: Supplementary file 1 — (a) Graphic representation of the first and second exon of gene LpCBP20 together with the gRNAs and primers used in this study. (b) Graphic representation of the first and second exons of the LpCRPK1 paralogs targeted in conjunction with the gRNAs and primers used. [file DataSheet1.pdf]

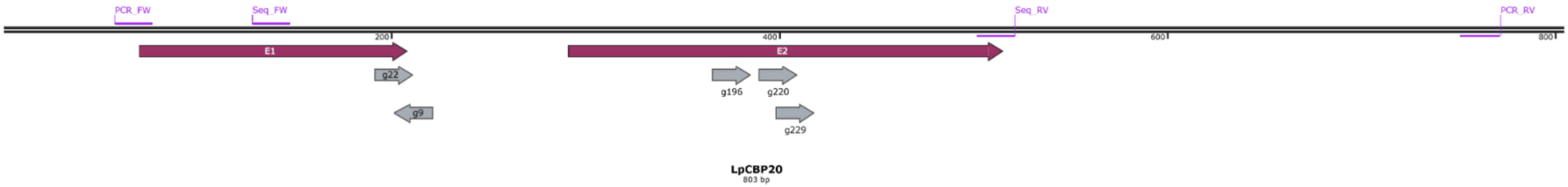

Supplementary file 1 a) Graphic representation of the first and second exon of the targeted gene (LpCBP20) together with the gRNAs and primers used in this study. The maroon arrows represent the two exons where the gRNAs aimed to induce indels. The location which the gRNAs targeted is indicated by the grey arrows. In purple, the image depicts the location which the PCR and sequencing primers targeted. This image was adapted from a picture generated with SnapGene® viewer software (from Dotmatics; available at snapgene.com)

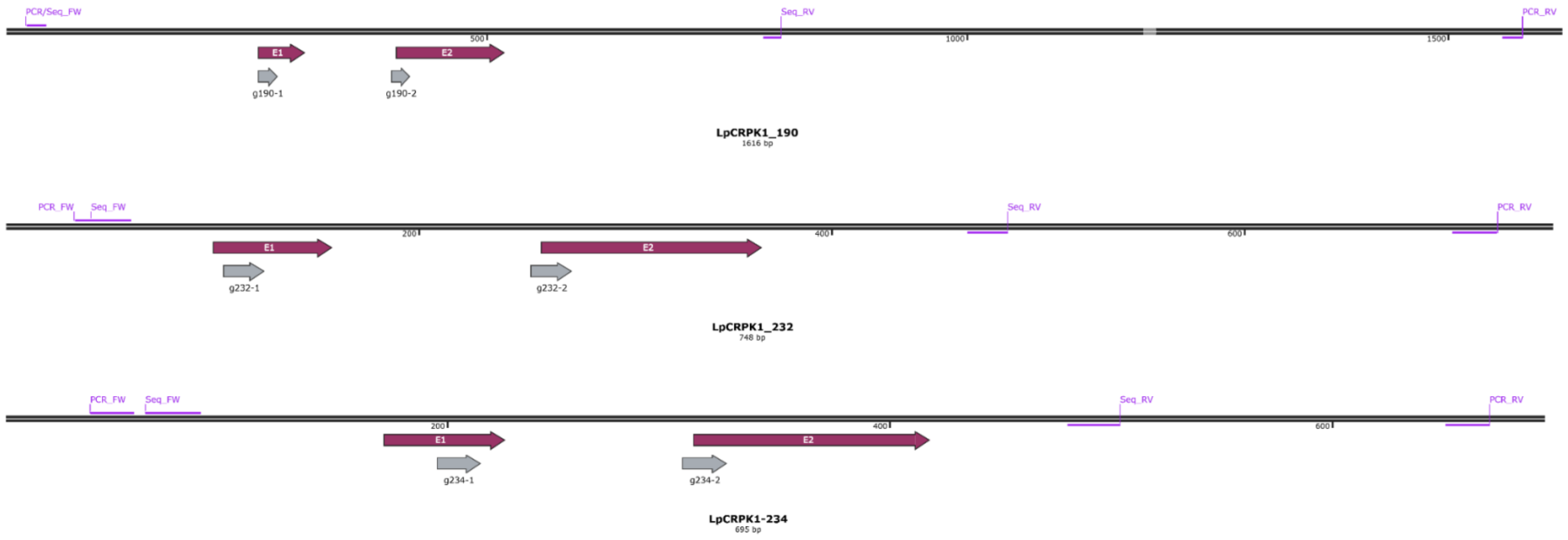

b) Graphic representation of the first and second exon of the targeted genes (LpCRPK1, paralogs Lp_chr7_0.1G7190, Lp_chr2_0G21232 and Lp_chr2_0G21234) together with the gRNAs and primers used in this study. The maroon arrows represent the two exons where the gRNAs aimed to induce indels. The location which the gRNAs targeted is indicated by the grey arrows. In purple, the image depicts the location which the PCR and sequencing primers targeted. This image was adapted from a picture generated with SnapGene® viewer software (from Dotmatics; available at snapgene.com)
