# Supplementary material for: Isolation and transformation of perennial ryegrass (Lolium perenne L.) protoplasts for the in vivo assessment of guide RNAs editing efficiency
Source: Front Plant Sci. 2026 Jan 16;16:1744085. doi: 10.3389/fpls.2025.1744085 (PMC12856575; doi:10.3389/fpls.2025.1744085)
Supplement: Supplementary file 2 — Sequences of gene LpCBP20 and LpCRPK1 paralogs containing location of primers, gRNAs and targeted exons. [file DataSheet2.pdf]

LpCBP20

```
5'   a t t t a c t c g t c c t c t t c c a t c c t c c t c c c t t t c t t t c g t a g a t a t   45
3'   t a a a t g a g c a g g a g a a g g t a g g a g g a g g g a a a g a a a g c a t c t a t a

                    PCR_FW
                    C G T G A G C A G A A G G G T T C A G A
     a g a a a a g t a a t t c g t g a g c a g a a g g g t t c a g a g a t t g g c c t a c c a   90
     t c t t t t c a t t a a g c a c t c g t c t t c c c a a g t c t c t a a c c g g a t g g t
                                                      E1

                                                                                  Seq_FW
                                                                                  T C G C T C G
     c c a a a g a c a t c a a a t t c c c c a a c t c c a a c t c c a a c t c t t c g c t c g   135
     g g t t t c t g t a g t t t a a g g g g t t g a g g t t g a g g t t g a g a a g c g a g c
                                      E1

       Seq_FW
     A C T C A A A A C C C T C
     a c t c a a a a c c c t c a c c t t c a a t a a g g c c t c t c g a c c t a g c g c g g c   180
     t g a g t t t t g g g a g t g g a a g t t a t t c c g g a g a g c t g g a t c g c g c c g
                                      E1

     c g c c g c a a c a a t g g c g t c c c t c t t c a a g g t a c g g a t c c c c c c g g a   225
     g c g g c g t t g t t a c c g c a g g g a g a a g t t c c a t g c c t a g g g g g g c c t
                       E1                                    PAM
                                g22
                          PAM                 g9

     a c c c t a g c c a a g c c t t c g a t t c g c c c c g c c t c t c c g c t c a t a c g g   270
     t g g g a t c g g t t c g g a a g c t a a g c g g g g c g g a g a g g c g a g t a t g c c

     c c c g c g c c t t t g c t t t t c c a g g a c c c g a g c a a g c t g t c g g t g t a c   315
     g g g c g c g g a a a c g a a a a g g t c c t g g g c t c g t t c g a c a g c c a c a t g
                                                    E2

     c g g g a c c g g c g g t t c a c c g g g a c g c a g g a g g a g t t c g a g g c g g c g   360
     g c c c t g g c c g c c a a g t g g c c c t g c g t c c t c c t c a a g c t c c g c c g c
                                      E2
```

ctgctcgcgtcgctgacggtgtatgtggggaacatgtccttctac 405
gacgagcgcagcgactgccacatacaccccttgtacaggaagatg

E2

g196 PAM g220

g229

agcacggaggagcaggcctacgagctattctcccgcgccggcgag 450
tcgtgcctcctcgtccggatgctcgataagagggcgcggccgctc

E2

g220 PAM PAM

g229

atcaagaagatcatcatgggactcgacaagaacaccaagaccccc 495
tagttcttctagtagtaccctgagctgttcttgtggttctggggg

E2

tgcggcttctgcttcgtactgtgcgtgcttctttccgtttcgtcc 540
acgccgaagacgaagcatgacacgcacgaagaaaggcaaagcagg

E2

AAGACGAAGCATGACACGCA

Seq_RV

ctcacggtttcatttatttagctcctcttatgttggatctggttc 585
gagtgccaaagtaaataaatcgaggagaatacaacctagaccaag

tgtagtatgccaacattctaggcaaccggcgttaccctaatttag 630
acatcatacggttgtaagatccgttggccgcaatgggattaaatc

atgcttgaaggatggctgtctagttctgattattgaggtccagat 675
tacgaacttcctaccgacagatcaagactaataactccaggtcta

gtattttgatcttgaaggatggctgtctagttctgattatttatt 720
cataaaactagaacttcctaccgacagatcaagactaataaataa

gacttaaattcgttggttgtgtgatgattgtgacttgtgagaggc 765
ctgaatttaagcaaccaacacactactaacactgaacactctccg

ACTGAACACTCTCCG

PCR_RV

aacctttgaattatgggattacaatgcgtgatgcaatt 3'
803
ttggaaacttaataccctaatgttacgcactacgttaa 5'

TTGGAA

PCR_RV

LpCRPK1_190

PCR/Seq_FW
TCATATCATGAGATTCGCCTTC

5' taggagatcttctagaaagatcatatcatgagattcgccttcttg 45
3' atcctctagaagatctttctagtatagtactctaagcggaagaac

aaaattggcaattcttcaaatccagaagacagatagaattccagg 90
ttttaaccgttaagaagtttaggtcttctgtctatcttaaggtcc

attatactttggcaaagacttctatgcagaattcttccagatcat 135
taatatgaaaccgtttctgaagatacgtcttaagaaggtctagta

ttttgccatactatatttcctatcagaagggaaaactnttttttt 180
aaaacggtatgatataaaggatagtcttcccttttganaaaaaaa

atgccgctctttctttctcagaattcagctccaatttagtcagta 225
tacggcgagaaagaaagagtcttaagtcgaggttaaatcagtcat

ctgtcgggatccacgatttgacttctgagttacaaacatggcttc 270
gacagccctaggtgctaaactgaagactcaatgtttgtaccgaag
E1
g190-1

ttgctttatgtgtggaaaggacatcaaagaaactattgaaggtga 315
aacgaaatacacacctttcctgtagtttctttgataacttccact
E1
g190-1
PAM

gtgtttcatttctttgctcagttttataacaamttctacttacag 360
cacaaagtaaagaaacgagtcaaaatattgttkaagatgaatgtc

aaagacgggcatcattctgattcacttgcatttgctttcttgtag 405
tttctgcccgtagtaagactaagtgaacgtaaacgaaagaacatc
g190-2

ctccaggaggaaataaggtgagggtttttttcttatagtgagctga 450
gaggtcctcctttattccactcccaaaaaagaatatcactcgact
E2
g190-2
PAM

ggaagggtacacatgattttagtgaggcaaacaagattggcgaag 495
ccttcccatgtgtactaaaatcactccgtttgttctaaccgcttc
E2

```
gcggttttggttctgtgttcagggtaattttctcaagycattgkt
cgccaaaaccaagacacaagtcccattaaaagagttcrgtaacma   540
E2

cacttcaaaagtccatcrcattgaactgtcttaactaatatttat
gtgaagttttcaggtagygtaacttgacagaattgattataaata   585

tttgtttgatcagggaaggcttaaagatggcacagttgtagcggt
aaacaaactagtcccttccgaatttctaccgtgtcaacatcgcca   630

aaaggtgctttctgccatttcgaagcaaggcataaaagagttttt
tttccacgaaagacggtaaagcttcgttccgtattttctcaaaaa   675

taccgaacttacagcaatttctgatattgtgcacgaaaacctgat
atggcttgaatgtcgttaaagactataacacgtgcttttggacta   720

caccttagtcggttgctgcgctgagggatcccacaggattcttgt
gtggaatcagccaacgacgcgactccctagggtgtcctaagaaca   765

atacaactatcttgagaacaacagccttgcacatacactgctagg
tatgttgatagaactcttgttgtcggaacgtgtatgtgacgatcc   810
GTCGGAACGTGTATGTGACG
Seq_RV

tgcctaacatgtccttcatctttcatgygaggcttcaataactat
acggattgtacaggaagtagaaagtacrctccgaagttattgata   855

cttacttaagtatgtttcatggtattgcaggaaaaggttatagca
gaatgaattcatacaaagtaccataacgtccttttccaatatcgt   900

gcatccggttgaactggagagttcgtgttaaaattgctgtaggca
cgtaggccaacttgacctctcaagcacaattttaacgacatccgt   945

ttgcccatgggcttgcgtttcttcatgaagaaatccgtcctccca
aacgggtacccgaacgcaaagaagtacttctttaggcaggagggt   990

taatccaccgggacataaaggcragcaatattcttctcgacaagg
attaggtggccctgtatttccgytcgttataagaagagctgttcc   1035

atctcacccccaaaatttcygattttgggttggcaaggctcctcc
tagagtgggggttttaaagrctaaaacccaaccgttccgaggagg   1080

ctccaaacgcaactcatgtgagcacctgtgtcgcaggtacaatgt
gaggtttgcgttgagtacactcgtggacacagcgtccatgttaca   1125
```

```
aagtataaccttgttagcgttttgtttgggcattagactatattt
                                                1170
ttcatattggaacaatcgcaaaacaaacccgtaatctgatataaa

cattcttatctctnnnnnnnnnnnnnttyccccctttctgtcaga
                                                1215
gtaagaatagagannnnnnnnnnnnnaarggggggaaagacagtct

ggataccttgctcctgaatatgcagttagaggtcaagtgacaaag
                                                1260
cctatggaacgaggacttatacgtcaatctccagttcactgtttc

aagtcagatatttatagttttggtgttctgctcttggaaatcgtt
                                                1305
ttcagtctataaatatcaaaaccacaagacgagaacctttagcaa

agtggcaggtgtaaccacaataatagattgccttatgaagatcaa
                                                1350
tcaccgtccacattggtgttattatctaacggaatacttctagtt

tttctgctcgagagggtaagctmtatcttaattcgatgttcamta
                                                1395
aaagacgagctctcccattcgakatagaattaagctacaagtkat

aacctacgctttattttgagaccgcagttgtactcctcyatttcc
                                                1440
ttggatgcgaaataaaactctggcgtcaacatgaggagrtaaagg

attatgaagtwaacatttaggtgttagctcgaacatacctgataa
                                                1485
taatacttcawttgtaaatccacaatcgagcttgtatggactatt

gatacttggctacygtrtagttcatctttttcgctttctttctcc
                                                1530
ctatgaaccgatgrcayatcaagtagaaaaagcgaaagaaagagg

ttttctctcctgtttttaccttgtagccttgctacgygttgttct
                                                1575
aaaagagaggacaaaaatggaacatcggaacgatgcrcaacaaga
                                  GGAACGATGCrCAACAAGA
                                        PCR_RV

ccacccgctttctttgcggtttacttctaatatacaacaca     3'
                                              1616
ggtgggcgaaagaaacgccaaatgaagattatatgttgtgt     5'
GG
PCR_RV
```

LpCRPK1_232

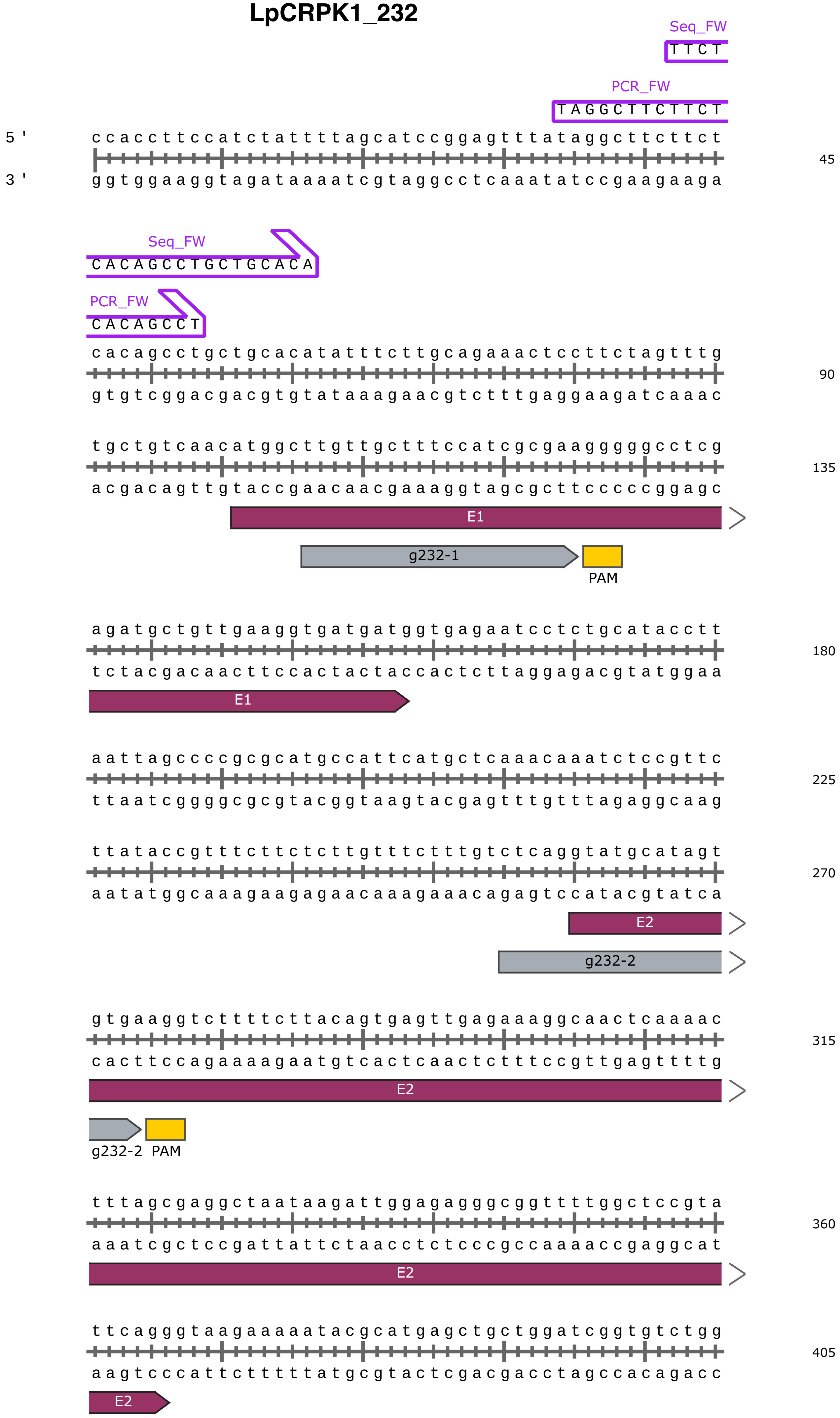
Seq_FW
TTCT
PCR_FW
TAGGCTTCTTCT
5 ' ccaccttccatctattttagcatccggagtttataggcttcttct
45
3 ' ggtggaaggtagataaaatcgtaggcctcaaatatccgaagaaga
Seq_FW
CACAGCCTGCTGCACA
PCR_FW
CACAGCCT
cacagcctgctgcacatatttcttgcagaaactccttctagtttg
90
gtgtcggacgacgtgtataaagaacgtctttgaggaagatcaaac
tgctgtcaacatggcttgttgctttccatcgcgaagggggcctcg
135
acgacagttgtaccgaacaacgaaaggtagcgcttcccccggagc
E1
g232-1
PAM
agatgctgttgaaggtgatgatggtgagaatcctctgcatacctt
180
tctacgacaacttccactactaccactcttaggagacgtatggaa
E1
aattagccccgcgcatgccattcatgctcaaacaaatctccgttc
225
ttaatcggggcgcgtacggtaagtacgagtttgtttagaggcaag
ttataccgtttcttctcttgtttctttgtctcaggtatgcatagt
270
aatatggcaaagaagagaacaaagaaacagagtccatacgtatca
E2
g232-2
gtgaaggtcttttcttacagtgagttgagaaaggcaactcaaaac
315
cacttccagaaaagaatgtcactcaactctttccgttgagttttg
E2
g232-2
PAM
tttagcgaggctaataagattggagagggcggttttggctccgta
360
aaatcgctccgattattctaacctctcccgccaaaaccgaggcat
E2
ttcagggtaagaaaaatacgcatgagctgctggatcggtgtctgg
405
aagtcccattctttttatgcgtactcgacgacctagccacagacc
E2

atgtcctagcgccttctgctaacatttcattttgcttcatcaggg 450
tacaggatcgcggaagacgattgtaaagtaaaacgaagtagtccc

caagctcaaagacggcacgtttattgcagtgaaggtgttgtccgc 495
gttcgagtttctgccgtgcaaataacgtcacttccacaacaggcg
GTGCAAATAACGTCACTTCC
Seq_RV

gacttcgaggcaaggcgttggagagttcttaactgaacttacggc 540
ctgaagctccgttccgcaacctctcaagaattgacttgaatgccg

aatttctgacattaagcatgaaaacctggtcacgcttatgggttg 585
ttaaagactgtaattcgtacttttggaccagtgcgaatacccaac

ctctgctgaagggtcccataggatcctcgtttacaattatcttga 630
gagacgacttcccagggtatcctaggagcaaatgttaatagaact

gaataacagccttcaacagacattgctaggtaacgctttgatcta 675
cttattgtcggaagttgtctgtaacgatccattgcgaaactagat

ctgaacatgtctggggatactcagatccttcacaggttaggtagc 720
gacttgtacagacccctatgagtctaggaagtgtccaatccatcg
AGGAAGTGTCCAATCCATCG
PCR_RV

atacttacagatgttctggatgtgctat 3'
748
tatgaatgtctacaagacctacacgata 5'
TA
PCR_RV

LpCRPK1_234

PCR_FW
C T C T A G G

5 ' c a g a t c t t c c g g a t g g c t c g a g y t t k t c a g c a a g a n n t c t c t a g g 45
3 ' g t c t a g a a g g c c t a c c g a g c t c r a a m a g t c g t t c t n n a g a g a t c c

PCR_FW — C T A A T G G G T T G C C
Seq_FW — A C C T C T C T T T T G G A T C A T C T A T T G T

c t a a t g g g t t g c c a a t a c a c c t c t c t t t t g g a t c a t c t a t t g t t g 90
g a t t a c c c a a c g g t t a t g t g g a g a g a a a a c c t a g t a g a t a a c a a c

c a a t g g a a g t a t a g t t a t a t t g t t t t c t g a t a t c c t a c t a c a t a t 135
g t t a c c t t c a t a t c a a t a t a a c a a a a g a c t a t a g g a t g a t g t a t a

a t c t c t t g c a g g a g t t c c t t c c a a t c t a c c a t a a a c a t g g a t t g c 180
t a g a g a a c g t c c t c a a g g a a g g t t a g a t g g t a t t t g t a c c t a a c g

E1

t g c t t t a t g t t c c g a a a g a g a t c t c a g c c t g t t g a a g g c g a t g a t 225
a c g a a a t a c a a g g c t t t c t c t a g a g t c g g a c a a c t t c c g c t a c t a

E1

g234-1 — PAM

g g t g a g t a c c t t c t g c a t a t c t a a g c a t g c c t g c t c a a a c a a t t t 270
c c a c t c a t g g a a g a c g t a t a g a t t c g t a c g g a c g a g t t t g t t a a a

E1

t c c c t g c t t a t a c g a t t t c t t c t g c t g t t c t t t t t t c t c a g a t g t 315
a g g g a c g a a t a t g c t a a a g a a g a c g a c a a g a a a a a a g a g t c t a c a

E2

g234-2

a c a c a g t g t g a a g g t c t t t t c t t a c a a c g a g t t g a g g a a g g c a a c 360
t g t g t c a c a c t t c c a g a a a a g a a t g t t g c t c a a c t c c t t c c g t t g

E2

g234-2 — PAM

t c t a g a t t t c a g c g g g g c a a a c a a g a t t g g a g a g g g t g g t t t t g g 405
a g a t c t a a a g t c g c c c c g t t t g t t c t a a c c t c t c c c a c c a a a a c c

E2

ctccgtattcagggtaaatatgcatgagctgctacctacttcatc 450
gaggcataagtcccatttatacgtactcgacgatggatgaagtag

E2

agtgtctaatagtcttgacattatcaaacacctttcgctaacatt 495
tcacagattatcagaactgtaatagtttgtggaaagcgattgtaa

GGAAAGCGATTGTAA
Seq_RV

tcattttgcttcatcagggaatgctcaaagacggcagattagttg 540
agtaaaacgaagtagtcccttacgagtttctgccgtctaatcaac

AGTAAAACG
Seq_RV

cagtgaaggtgctctcagcgacttcaaggcaaggtgtccgagagt 585
gtcacttccacgagagtcgctgaagttccgttccacaggctctca

tcttaactgaacttacggcaatttctgacatcaagcatgaaaacc 630
agaattgacttgaatgccgttaaagactgtagttcgtacttttgg

tggtcacgcttatcggctgctgtgctgaagggtcccataggatcc 675
accagtgcgaatagccgacgacacgacttcccagggtatcctagg

CACGACTTCCCAGGGTATCC
PCR_RV

tcgtttacaattaccttgag 3'
695
agcaaatgttaatggaactc 5'
