# Supplementary material for: Isolation and transformation of perennial ryegrass (Lolium perenne L.) protoplasts for the in vivo assessment of guide RNAs editing efficiency
Source: Front Plant Sci. 2026 Jan 16;16:1744085. doi: 10.3389/fpls.2025.1744085 (PMC12856575; doi:10.3389/fpls.2025.1744085)
Supplement: Supplementary file 3 — Map of the pHSE401/EGFP plasmid. [file DataSheet3.pdf]

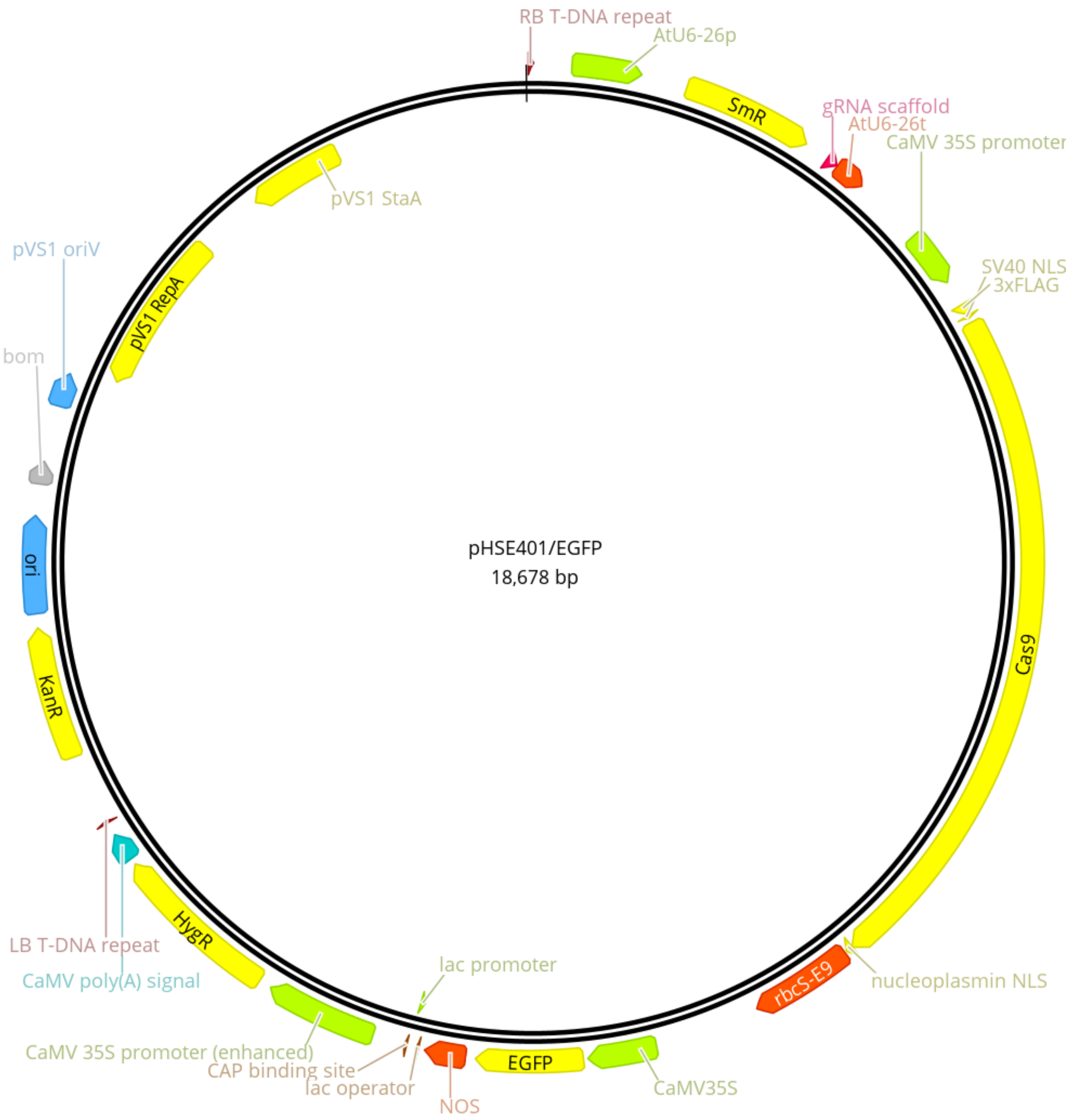

Supplementary file 3. Map of the pHSE401/EGFP plasmid. This was the backbone used to generate vectors p196 and p229. Vector p196 had the sequence coding for gRNA 196 inserted at the "gRNA scaffold" region, while p229 had the sequence for gRNA 229 inserted at this same region.
