# Supplementary material for: Isolation and transformation of perennial ryegrass (Lolium perenne L.) protoplasts for the in vivo assessment of guide RNAs editing efficiency
Source: Front Plant Sci. 2026 Jan 16;16:1744085. doi: 10.3389/fpls.2025.1744085 (PMC12856575; doi:10.3389/fpls.2025.1744085)
Supplement: Supplementary file 4 — (a) Map of the transformation vector pCBP20_5g. (b) Graphical representation of the transcriptional unit encoding the five gRNAs in the pCBP20_5g plasmid. [file DataSheet4.pdf]

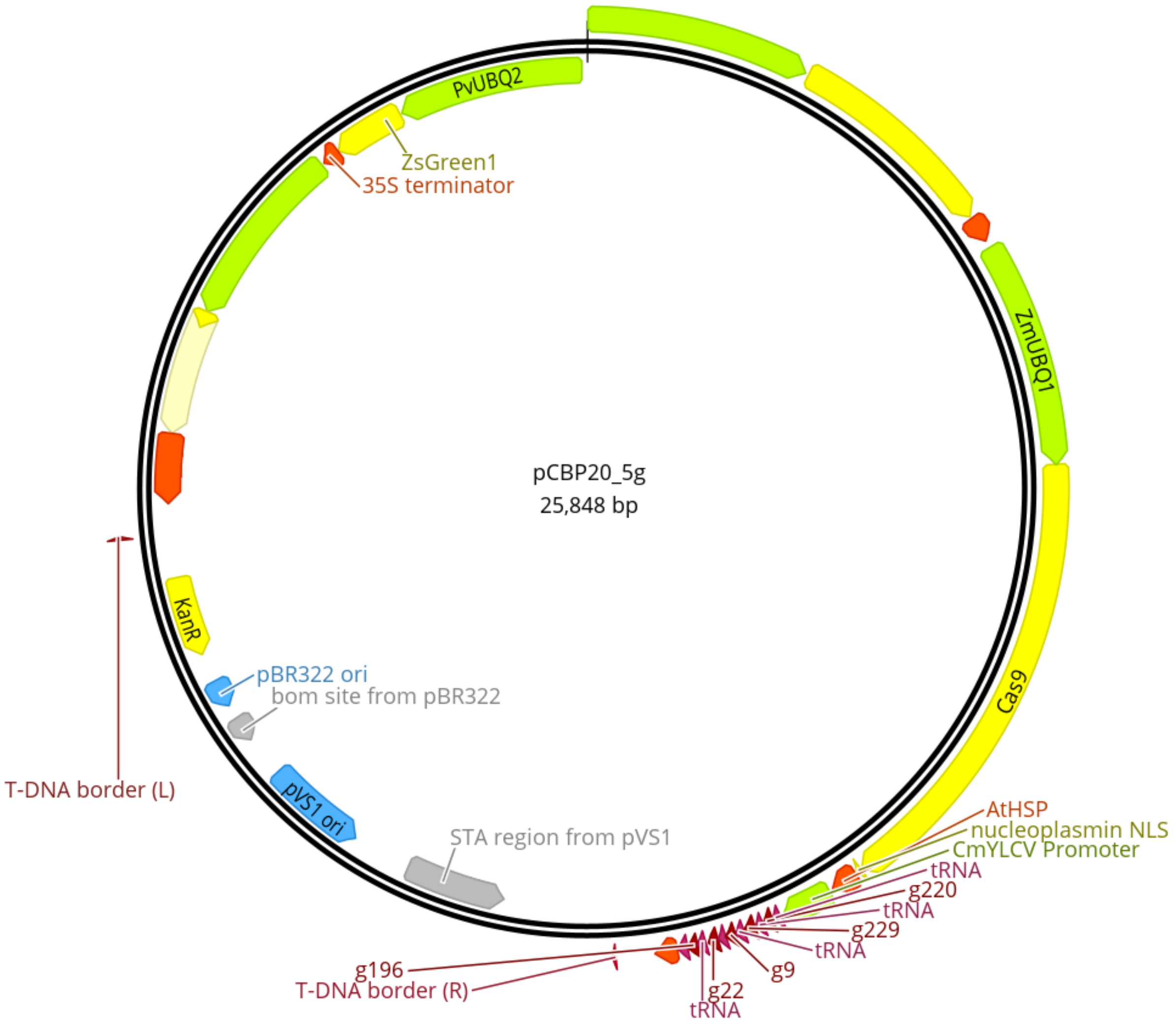

Supplementary file 4. a) Map of the transformation vector pCBP20_5g. Inside the T-DNA region, the plasmid has transcriptional units encoding the Cas9 nuclease, five different gRNAs with tRNAs as spacers, and a ZsGreen fluorescent protein.

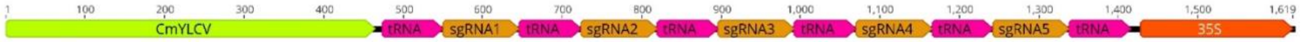

b) Graphical representation of the transcriptional unit encoding the five gRNAs in the pCBP20_5g plasmid. tRNAs are present as spacers. The cassette is under the control of the CmYLCV promoter and has as terminator CaMV35S.
