# Supplementary material for: Isolation and transformation of perennial ryegrass (Lolium perenne L.) protoplasts for the in vivo assessment of guide RNAs editing efficiency
Source: Front Plant Sci. 2026 Jan 16;16:1744085. doi: 10.3389/fpls.2025.1744085 (PMC12856575; doi:10.3389/fpls.2025.1744085)
Supplement: Supplementary file 5 — (a) Map of the transformation vector piCas9_CRPK1. (b) Graphical representation of the transcriptional unit encoding the six gRNAs present in piCas9_CRPK1. [file DataSheet5.pdf]

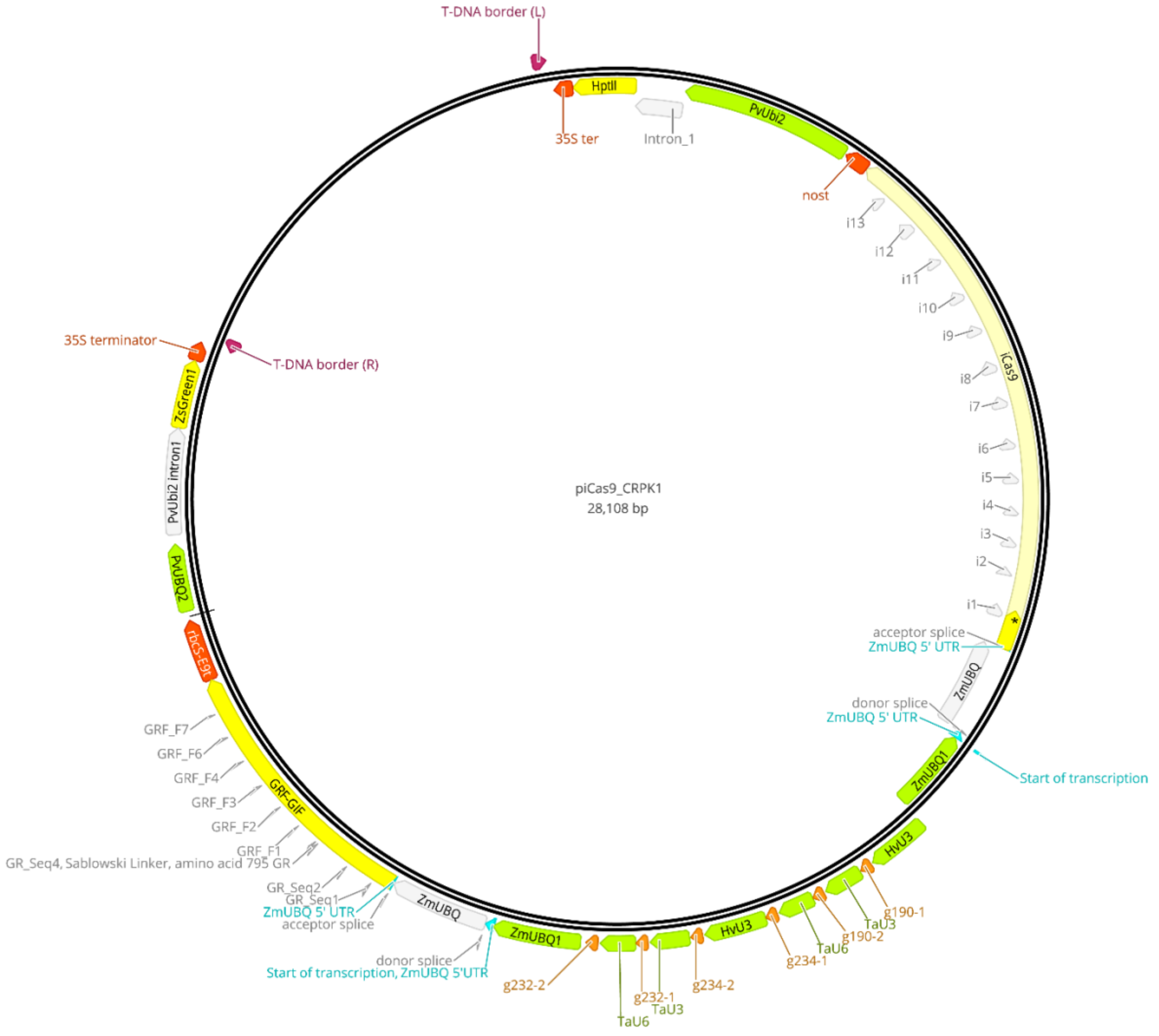

Supplementary file 5. a) Map of the transformation vector piCas9_CRPK1. Inside the T-DNA region, the plasmid has transcriptional units encoding the Cas9 nuclease, six different gRNAs, a ZsGreen fluorescent protein and a chimeric GRF-GIF protein.

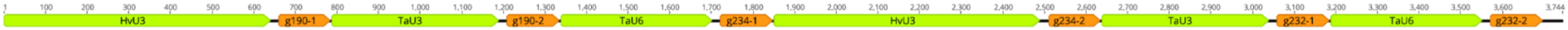

b) Graphical representation of the transcriptional unit encoding the six gRNAs in the piCas9_CRPK1 plasmid. Each gRNA is under the control of a different promoter (in green).
