# Supplementary material for: Isolation and transformation of perennial ryegrass (Lolium perenne L.) protoplasts for the in vivo assessment of guide RNAs editing efficiency
Source: Front Plant Sci. 2026 Jan 16;16:1744085. doi: 10.3389/fpls.2025.1744085 (PMC12856575; doi:10.3389/fpls.2025.1744085)
Supplement: Supplementary file 6 — (a) Table containing the primers used in this study. (b) Table containing the gRNAs used in this study. [file DataSheet6.pdf]

Supplementary file 6. a) Table containing the primers used in this study.

**Primer name**	**Sequence (5'-3')**
LpCBP20_PCR-FW	CGTGAGCAGAAGGGTTCAGA
LpCBP20_PCR-RV	TGACTTGTGAGAGGCAACCTT
LpCBP20_Seq-FW	TCGCTCGACTCAAAACCCTC
LpCBP20_Seq-RV	TTCTGCTTCGTACTGTGCGT
LpCRPK1-190_PCR/Seq-FW	CCATATCATGAGATTCGCCTTC
LpCRPK1-190_PCR-RV	CCTTGCTACGCGTTGTTCTCC
LpCRPK1-190_Seq-RV	CAGCCTTGCACATACACTGC
LpCRPK1-232_PCR-FW	TAGGCCTCTTCTCACAGCCT
LpCRPK1-232_PCR-RV	TCCTTCACAGGTTAGGTTAGCA
LpCRPK1-232_Seq-FW	TTCTCACAGCCTGCTGCATA
LpCRPK1-232_Seq-RV	CACGTTTGTTGCGGTGAAGG
LpCRPK1-234_PCR-FW	CTCTAGGCTAATGGGTTGCC
LpCRPK1-234_PCR-RV	GTGCTGAAGGGTCCCATAGG
LpCRPK1-234_Seq-FW	ACCTCTCTTTTGGATCATCTATTGT
LpCRPK1-234_Seq-RV	CCTTTCGCTAACATTTCATTTTGC

Supplementary file 6. b) Table containing the gRNAs used in this study.

**Guide name**	**Sequence (5'-3')**
g22	TGGCGTCCCTCTTCAAGGTA
g9	CTTCAAGGTACGGATCCCCC
g196	CGCGTCGCTGACGGTGTATG
g220	GAACATGTCCTTCTACAGCA
g229	CTTCTACAGCACGGAGGAGC
g190_1	ATGGCTTCTTGCTTTATGTG
g190_2	TGTAGCTCCAGGAGGAAATA
g234_1	AAGAGATCTCAGCCTGTTGA
g234_2	CTCAGATGTACACAGTGTGA
g232_1	TTGTTGCTTTCCATCGCGAA
g232_2	CTCAGGTATGCATAGTGTGA
