# Supplementary material for: Isolation and transformation of perennial ryegrass (Lolium perenne L.) protoplasts for the in vivo assessment of guide RNAs editing efficiency
Source: Front Plant Sci. 2026 Jan 16;16:1744085. doi: 10.3389/fpls.2025.1744085 (PMC12856575; doi:10.3389/fpls.2025.1744085)
Supplement: Supplementary file 7 — Images showing the amplification of the PCR products used for sequencing and subsequent analysis for indel assessment with TIDE. [file DataSheet7.pdf]

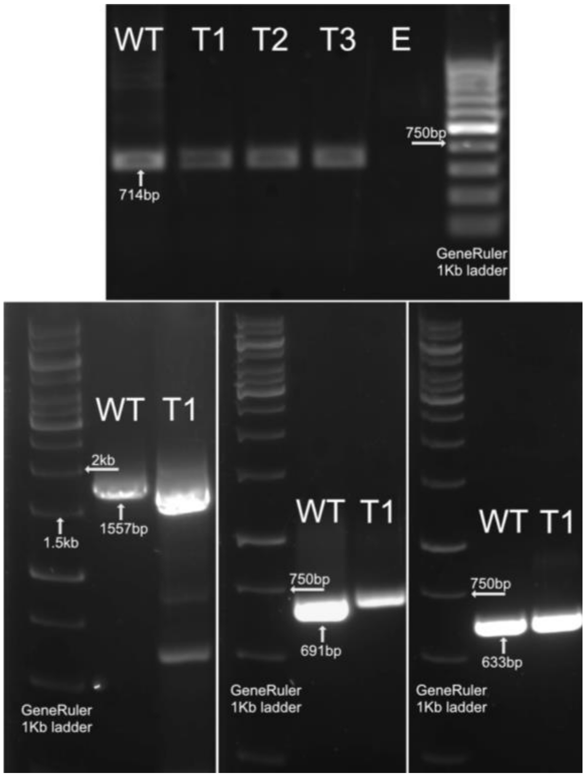

Supplementary file 7 Images showing the PCR amplification of products used for Sanger-sequencing and TIDE decomposition-analysis. The top panel shows the PCR products from amplifying a region of the *LpCBP20* gene from genomic DNA of non-transformed cells (WT) and protoplasts transformed using the pCBP20_5g vector (T1-3), with an empty well (E) next to the ladder. The bottom panels portray the amplification of a region of the *LpCRPK1* gene. From left to right, the images show the amplification of targeted regions of paralogs 190, 232 and 234. The images contain products from genomic DNA of non-transformed protoplasts (WT) and from cells transformed with the piCas9_CRPK1 vector (T1). All amplifications were done using ThermoFisher's Phusion™ High-Fidelity DNA Polymerase. In all pictures the used ladder was ThermoFisher's GeneRuler 1 kb DNA Ladder. The horizontal arrows denote the length of the ladder fragments closer to the amplified PCR products, below which vertical arrows indicate the length of the amplified fragments from protoplasts genomic DNA.
