# Supplementary material for: Isolation and transformation of perennial ryegrass (Lolium perenne L.) protoplasts for the in vivo assessment of guide RNAs editing efficiency
Source: Front Plant Sci. 2026 Jan 16;16:1744085. doi: 10.3389/fpls.2025.1744085 (PMC12856575; doi:10.3389/fpls.2025.1744085)
Supplement: Supplementary file 8 — (a) Representation of the indel frequencies of one editing event using the pCBP20_5g transformation vector. (b) Representation of the indel frequencies of one editing event when targeting paralogs LpCRPK1–190 and LpCRPK1–232 using the piCas9_CRPK1 transformation vector. [file DataSheet8.pdf]

frontiers

Exon 1 - gRNA_9 (R^2 = 0.91, pvalue <0.001)

WT 69.8% GCCGCAACAATGGCGTCCCTCTT¦CAAGGTACGGATCCCCCGGAACCCTA

-2 3.2% GCCGCAACAATGGCGTCCCTCTT¦– – AGGTACGGATCCCCCGGAACCCTA

-4 10.1% GCCGCAACAATGGCGTCCCTCTT¦– – – – GTACGGATCCCCCGGAACCCTA

Exon 1 - gRNA_22 (R^2 = 0.91, pvalue <0.001)

WT 70.6% GCCGCAACAATGGCGTCCCTCTTCAAG¦GTACGGATCCCCCGGAACCCTA

-4 10.1% GCCGCAACAATGGCGTCCCTCTT– – – – ¦GTACGGATCCCCCGGAACCCTA

Exon 2 - gRNA_196 (R^2 = 0.93, pvalue <0.001)

WT 72.6% CGGCGCTGCTCGCGTCGCTGACGGTGT¦ATGTGGGGAACATGTCCTTCTAC

-4 14.4% CGGCGCTGCTCGCGTCGCTGACG– – – – ¦ATGTGGGGAACATGTCCTTCTAC

+5 2.6% CGGCGCTGCTCGCGTCGCTGACGGTGT**NNNNN**¦ATGTGGGGAACATGTCCTTCTAC

Exon 2 - gRNA_220 (R^2 = 0.93, pvalue <0.001)

WT 69.9% TGTATGTGGGGAACATGTCCTTCTACA¦GCACGGAGGAGCAGGCCTACGAG

-4 15.5% TGTATGTGGGGAACATGTCCTTC– – – – ¦GCACGGAGGAGCAGGCCTACGAG

+4 1.9% TGTATGTGGGGAACATGTCCTTCTACA**NNNN**¦GCACGGAGGAGCAGGCCTACGAG

+5 2.6% TGTATGTGGGGAACATGTCCTTCTACA**NNNNN**¦GCACGGAGGAGCAGGCCTACGAG

Exon 2 - gRNA_229 (R^2 = 0.92, pvalue <0.001)

WT 68.5% GGAACATGTCCTTCTACAGCACGGAGG¦AGCAGGCCTACGAGCTATTCTCC

-4 15% GGAACATGTCCTTCTACAGCACG– – – – ¦AGCAGGCCTACGAGCTATTCTCC

+4 2.1% GGAACATGTCCTTCTACAGCACGGAGG**NNNN**¦AGCAGGCCTACGAGCTATTCTCC

+5 2.8% GGAACATGTCCTTCTACAGCACGGAGG**NNNNN**¦AGCAGGCCTACGAGCTATTCTCC

Supplementary file 8. a) Indels frequencies of the transformation using the pCBP20_5g plasmid targeting *LpCBP20*. The image above is a representation of the distribution of indel frequencies calculated by TIDE for one of the transformation events. The orange nucleotides denote the PAM region next to the gRNAs, which are marked in green. The vertical dashes represent the cleaving point of the Cas9 present in the transformation vector. Highlighted regions point deletions (dashes) or insertions (**N**) predicted by the deconvolution program. All the sequences are in the 5' - 3' orientation. It should be mentioned that guide 9 is in the antisense direction.

Exon 1 - gRNA_190-1 (R^2 = 0.95, pvalue <0.001)

WT 72.5% GTTACAAACATGGCTTCTTGCTTTAT¦GTGTGGAAAGGACATCAAAGAAAC

-1 10.3% GTTACAAACATGGCTTCTTGCTTTA-¦GTGTGGAAAGGACATCAAAGAAAC

-3 6.3% GTTACAAACATGGCTTCTTGCTT---¦GTGTGGAAAGGACATCAAAGAAAC

+1 2.8% GTTACAAACATGGCTTCTTGCTTTAT**N**¦GTGTGGAAAGGACATCAAAGAAAC

Exon 2 - gRNA_190-2 (R^2 = 0.98, pvalue <0.001)

WT 72.5% TTGCTTTCTTGTAGCTCCAGGAGGAA¦ATAAGGTGAGGGTTTTTCTTATA

-1 9% TTGCTTTCTTGTAGCTCCAGGAGGA-¦ATAAGGTGAGGGTTTTTCTTATA

-3 8% TTGCTTTCTTGTAGCTCCAGGAG---¦ATAAGGTGAGGGTTTTTCTTATA

+1 2.9% TTGCTTTCTTGTAGCTCCAGGAGGA**N**¦ATAAGGTGAGGGTTTTTCTTATA

Exon 1 - gRNA_232-1 (R^2 = 0.98, pvalue <0.001)

WT 92.2% TCAACATGGCTTGTTGCTTTCCATCGC¦GAAGGGGGCCTCGAGATGCTGTT

-9 1.5% TCAACATGGCTTGTTGCT---------¦GAAGGGGGCCTCGAGATGCTGTT

+1 1.9% TCAACATGGCTTGTTGCTTTCCATCG**N**¦GAAGGGGGCCTCGAGATGCTGTT

Exon 2 - gRNA_232-2 (R^2 = 0.99, pvalue <0.001)

WT 93.2% GTTTCTTTGTCTCAGGTATGCATAGTG¦TGAAGGTCTTTTCTTACAGTGAG

+4 3.2% GTTTCTTTGTCTCAGGTATGCAT**NNNN**¦TGAAGGTCTTTTCTTACAGTGAG

b) Indels frequencies of the transformation using the piCas9_CRPK1 plasmid targeting paralogs *LpCRPK1-190* and *LpCRPK1-232*. The image above is a representation of the distribution of indel

frequencies calculated by TIDE for one of the transformation events. The orange nucleotides denote the PAM region next to the gRNAs, which are marked in green. The vertical dashes represent the cleaving point of the Cas9 present in the transformation vector. Highlighted regions point deletions (dashes) or insertions (**N**) predicted by the deconvolution program. All the sequences are in the 5’ - 3’ orientation.
